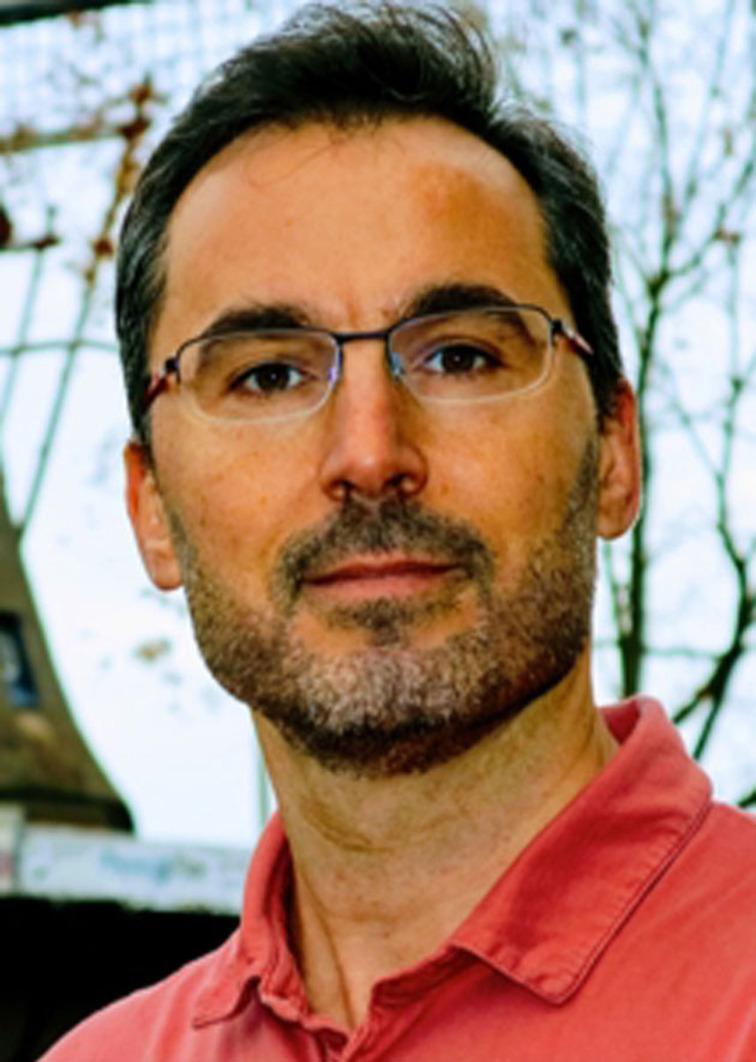# Cyclobutanes in Small‐Molecule Drug Candidates

**DOI:** 10.1002/cmdc.202200020

**Published:** 2022-03-29

**Authors:** Marnix R. van der Kolk, Mathilde A. C. H. Janssen, Floris P. J. T. Rutjes, Daniel Blanco‐Ania

**Affiliations:** ^1^ Institute for Molecules and Materials Radboud University Heyendaalseweg 135 6526 AJ Nijmegen The Netherlands

**Keywords:** Cyclobutanes, strained carbocycles, conformational restriction, pharmacophores, isosteres

## Abstract

Cyclobutanes are increasingly used in medicinal chemistry in the search for relevant biological properties. Important characteristics of the cyclobutane ring include its unique puckered structure, longer C−C bond lengths, increased C−C π‐character and relative chemical inertness for a highly strained carbocycle. This review will focus on contributions of cyclobutane rings in drug candidates to arrive at favorable properties. Cyclobutanes have been employed for improving multiple factors such as preventing *cis*/*trans*‐isomerization by replacing alkenes, replacing larger cyclic systems, increasing metabolic stability, directing key pharmacophore groups, inducing conformational restriction, reducing planarity, as aryl isostere and filling hydrophobic pockets.

## Graphical abstract

**The 3D structure** of cyclobutane opens opportunities for unique applications in medicinal chemistry. Improved synthetic methods and commercial availability now allow for more facile incorporation of this structural motif in small‐molecule drug candidates. Although its use has been increasing, it is still very much underused relative to other motifs in drug development. This review presents recent applications of the cyclobutane ring in small‐molecule drug development and the utility it can offer to medicinal chemists.

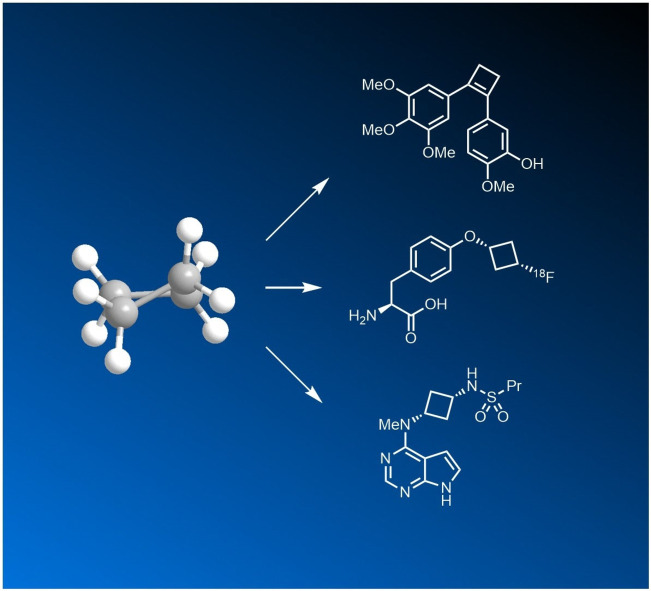

## 1 Introduction

### 1.1 Historical synopsis

**Figure 1:**
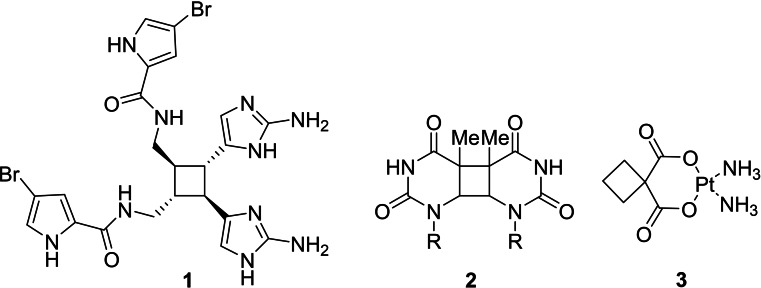
Cyclobutane **1**, isolated from Agelas sceptrum sea‐sponge, thymine dimer **2** (R indicates the rest of the DNA helix), and carboplatin (**3**).

Cyclobutane was first synthesized in 1907.^[1]^ It is a colorless gas with no biological properties as such. Cyclobutane rings, although relatively rare, do however occur in natural products, most of which are found in plant and marine species.^[2]^ The cyclobutane skeleton, present in sceptrins (e. g., **1**, also isolated in acetylated form and as HCl salt) from *Agelas sceptrum*, contributes to its antimicrobial properties (Figure 1).^[3]^

In addition, DNA bases can undergo [2+2] photodimerization upon UV irradiation and form cyclobutane pyrimidine dimers (**2**; Figure 1). Such DNA crosslinking can cause various adverse effects, often leading to skin cancer.^[4]^ Introduction of cyclobutane rings in pharmacologically active compounds is relatively new, but inorganic‐based cyclobutane drugs have been around for a longer time, most profoundly in a drug inducing cell death in cancer cells by crosslinking DNA. This drug, carboplatin (**3**), is widely used in severe forms of cancer, including ovarian, testicular, cervical, head and neck cancers.^[5]^ These compounds,^[6]^ as well as oxetanes^[7]^ and squaramide derivatives,^[8]^ have already been extensively reviewed elsewhere and will not be discussed in the current review.

Synthetic efforts towards cyclobutane derivatives have since then progressed, improving their usefulness in drug development. As the synthesis of cyclobutane building blocks has been extensively reviewed elsewhere,^[9]^ it will not be discussed in this review.

### 1.2 Properties of the cyclobutane ring

**Figure 2:**
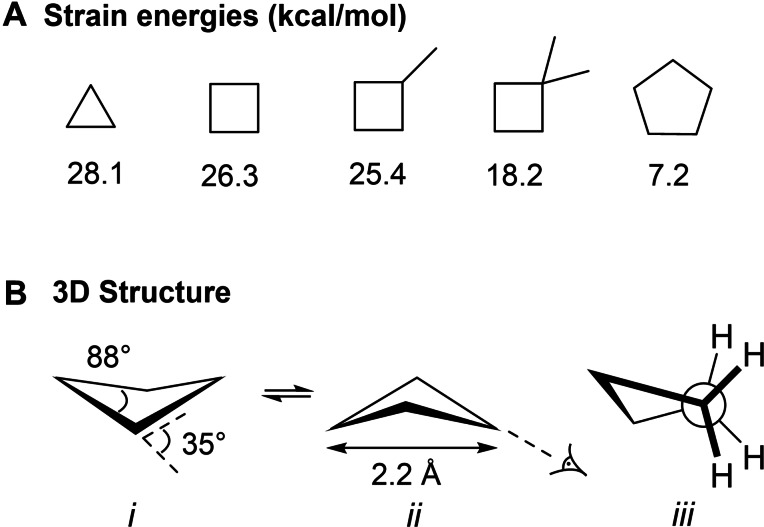
**A)** Strain energies of various cycloalkanes. **B)** 3D structure of cyclobutane (i and ii). Newman projection along the C−C bond. Its structure slightly deviates from eclipsed (iii).

The cyclobutane ring is the second most strained saturated monocarbocycle after cyclopropane with a strain energy of 26.3 kcal mol^−1^ (compared to 28.1 and 7.1 kcal mol^−1^ for cyclopropane and cyclopentane, respectively).[^10^, ^11^] Strikingly, the strain energy drastically lowers when the cyclobutane ring is mono‐ or di‐substituted with methyl groups because of the Thorpe–Ingold effect (Figure 2).^[10]^ With C−C bond lengths of 1.56 Å, the bond lengths are longer compared to ethane 1.54 Å. This lengthening is induced by 1,3 C−C non‐bonding repulsions, as the cross‐distance is only 2.22 Å. Comparatively, this interaction is not present in cyclopropane rings since every carbon is bound to two other carbon atoms, resulting in the shorter bond lengths of 1.53 Å.^[12]^ Cyclobutane adopts a folded structure, reducing its bond angle slightly to 88° compared to the expected 90°, which increases the angle strain, but at the same time relieves the torsional strain. This balance of energies leads to the puckered conformation as the energetically most favorable structure (Figure 2).^[13]^ As a result, the C−C bonds have slightly increased *p*‐character and the C−H bonds more *s*‐character.^[14]^

This increased *s*‐character is relatively subtle compared to cyclopropane. While cyclopropane readily reacts with sulfuric acid or undergoes bromination, cyclobutane does not.^[14]^ This reactivity puts cyclobutane in between the very reactive cyclopropane and relatively inert cyclopentane and other alkanes.

## 2 Influence on pharmacological activity

This perspective will focus on illustrating how cyclobutanes as regular and spirocyclic fused rings, as substituents, and with different substitution patterns (1,2‐disubstituted up to octasubstituted) can favorably contribute to drug properties of small molecules. Rather limited knowledge is present in current literature regarding medicinal chemistry properties of cyclobutane scaffolds such as metabolic stability or their use in medicinal chemistry in general. Therefore, this review is meant to fill the gap in literature, showing the implications and opportunities cyclobutane rings can offer to improve druglike properties of compounds in hit‐to‐lead development, using literature from the time period 2010–2020.

An often‐described property that is commonly introduced by the inclusion of a cyclobutane ring is conformational restriction. Flexible ligands can suffer from an entropic penalty upon binding because of the freezing of rotatable bonds in the binding pocket. For example, a flexible ethyl linker can be replaced by a 1,3‐disubstituted cyclobutane to limit the number of possible conformations. In addition, conformational restriction by introduction of a fused ring system can block metabolically labile sites.^[15]^ Furthermore, the use of saturated cyclobutane rings instead of planar aromatic rings correlates with stronger binding affinities because saturated molecules better complement spatial arrangements of target proteins. This saturation increase also correlates to higher water solubility and lower melting points, both of which are key to successfully develop a lead compound.^[16]^ Moreover, the cyclobutyl ring can be used to direct key pharmacophore groups,[^17^, ^18^, ^19^, ^20^, ^21^, ^22^] fill a hydrophobic pocket in the target enzyme,[^17^, ^22^, ^23^, ^24^, ^25^, ^26^, ^27^, ^28^, ^29^, ^30^] prevent *cis*/*trans*‐isomerization,[^31^, ^32^, ^33^] improve metabolic stability,[^34^, ^35^, ^36^] replace aromatic groups as an aryl isostere,^[37]^ conformationally restrict (part of) the molecule[^38^, ^39^, ^40^, ^41^] or reduce planarity.^[42]^

The use of cyclobutanes in current drugs is limited compared to the use of other recurring structural moieties. As of January 2021, there were at least 39 (pre)clinical drug candidates containing a cyclobutane ring (DrugBank 5.1 database search),^[43]^ some of which were discovered during the selected time and hence discussed in this review. The aim of this review is to provide an overview of different applications of cyclobutane rings in medicinal chemistry showing the positive effects this ring can have on the pharmacological properties of small molecules. The examples that follow in the next sections are ordered by disease area.

### 2.1 Cyclobutanes in anticancer compounds

**Figure 3:**
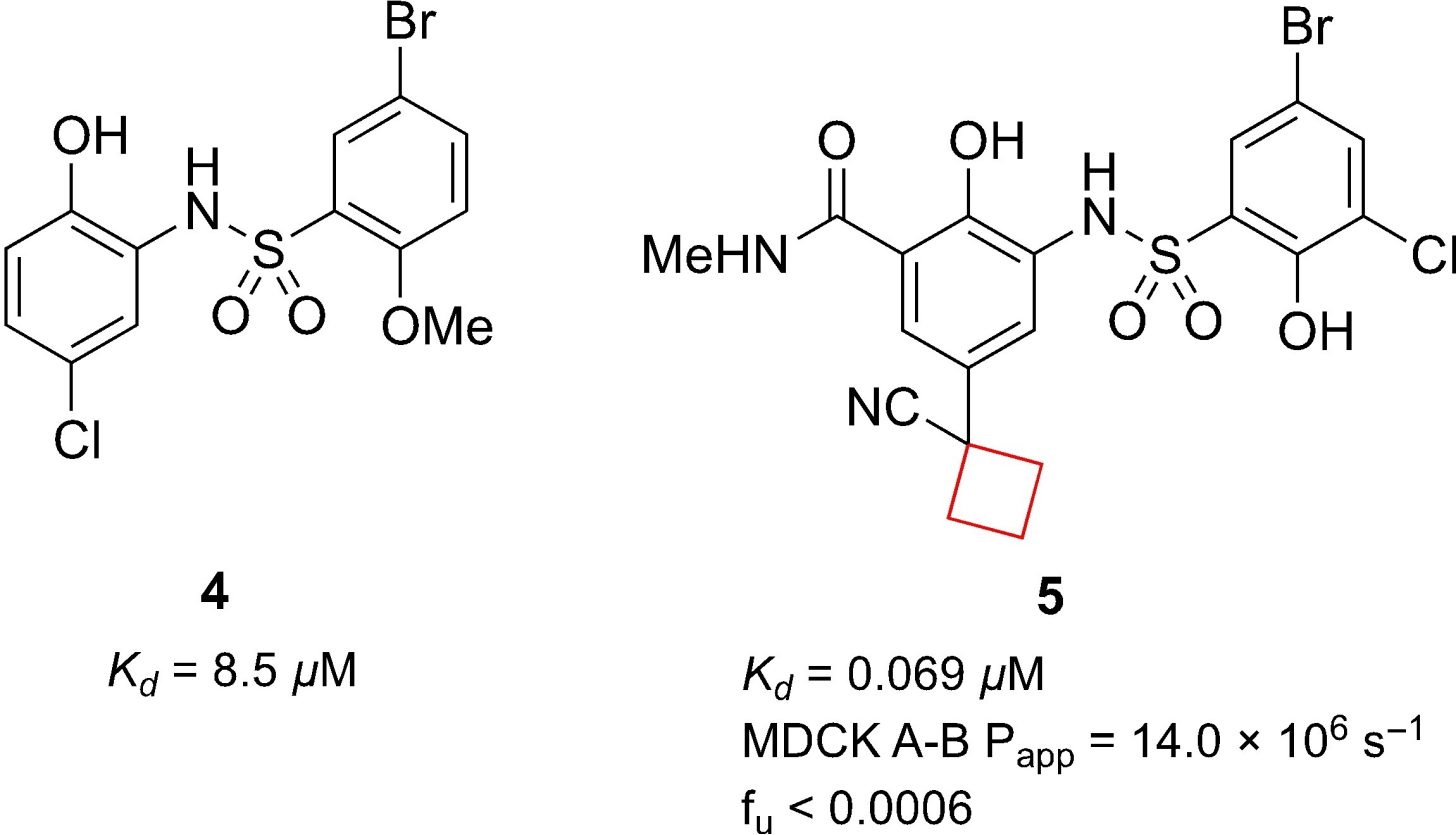
WDR inhibitors, HTS hit **4** and optimized lead compound **5**.

MYC genes are a family of oncogenes encoding transcription factors involved in the regulation of apoptosis, cell metabolism, genome instability, cell growth and proliferation. As a consequence, MYC overexpression is often observed in cancer cells.^[44]^ The interaction of MYC with WD repeat‐containing protein 5 (WDR5) is a key interaction in carcinogenesis and its interaction site has been recognized as a target for small‐molecule inhibitors.^[45]^ By high throughput screening (HTS), Macdonald et al.^[17]^ identified compound **4** as a hit showing inhibitory effects (Figure 3). Upon lead optimization, it demonstrated a high potency towards inhibition of the oncogenic function of MYC transcription factors. In this process, the cyclobutyl substituents (as well as other cycloalkanes) were originally installed on the phenolic ring as a tool for further growth along the edge of the WDR5 binding motif. It was, however, found that the cyclobutyl ring possessed optimal properties to complement the hydrophobic region of the binding pocket. It directs the nitrile group more towards the protein, with no observable directional interaction, but increased affinity nonetheless, which resulted in optimized structure **5** (Figure 3).

**Figure 4:**
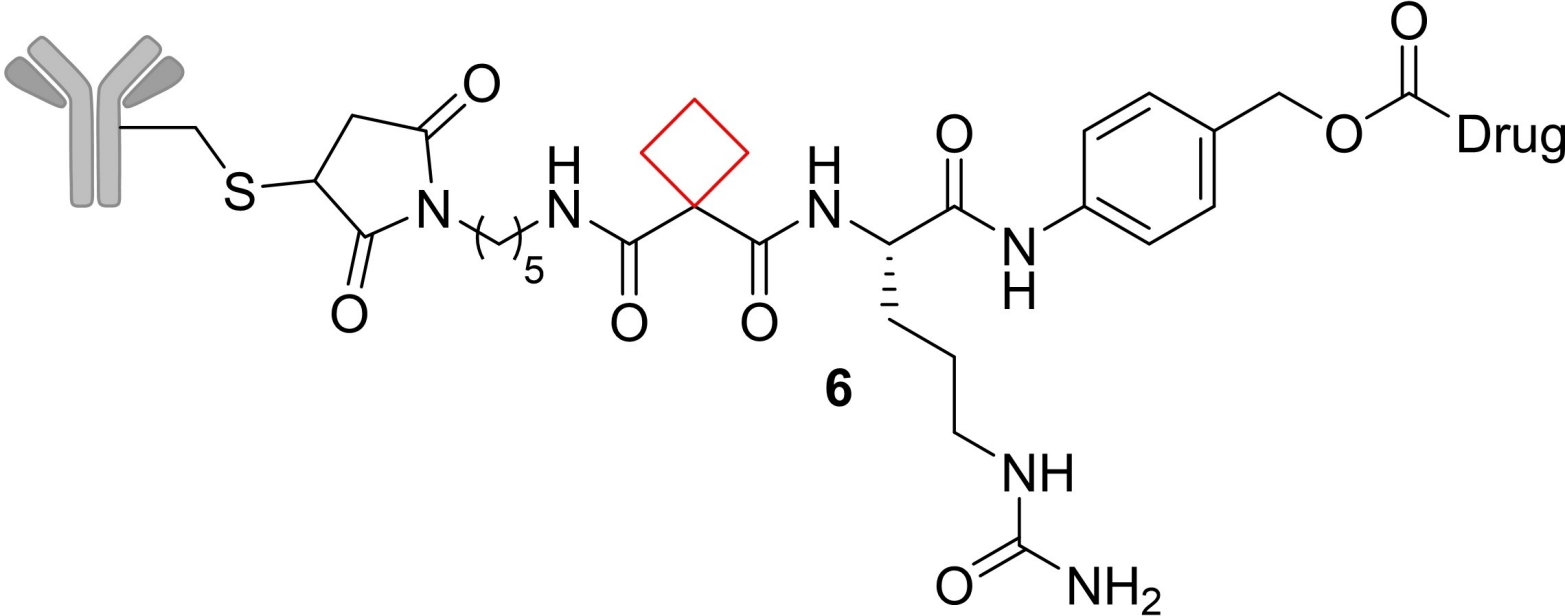
Cathepsin B *ADC **6***.

Cathepsin B is an enzyme of the lysosomal cysteine protease family.^[46]^ Overexpression of this enzyme is prevalent in invasive and metastatic cancers.^[47]^ In an effort to improve peptide linker stability of an antibody‐drug conjugate (ADC) that targets cancer cells where cathepsin B is active, Wei et al.^[23]^ installed a cyclobutyl ring replacing a valine residue. It was hypothesized by a computational similarity search that this moiety would fit best in the hydrophobic binding pocket of cathepsin B. After successfully synthesizing numerous candidates, including compound **6** (Figure 4), it appeared that this cyclobutane‐containing linker showed greater selectivity towards cathepsin B over other enzymes, increasing its selectivity towards tumor cells compared to valine‐citrulline linker systems that are already in clinical trials.^[48]^

**Figure 5:**
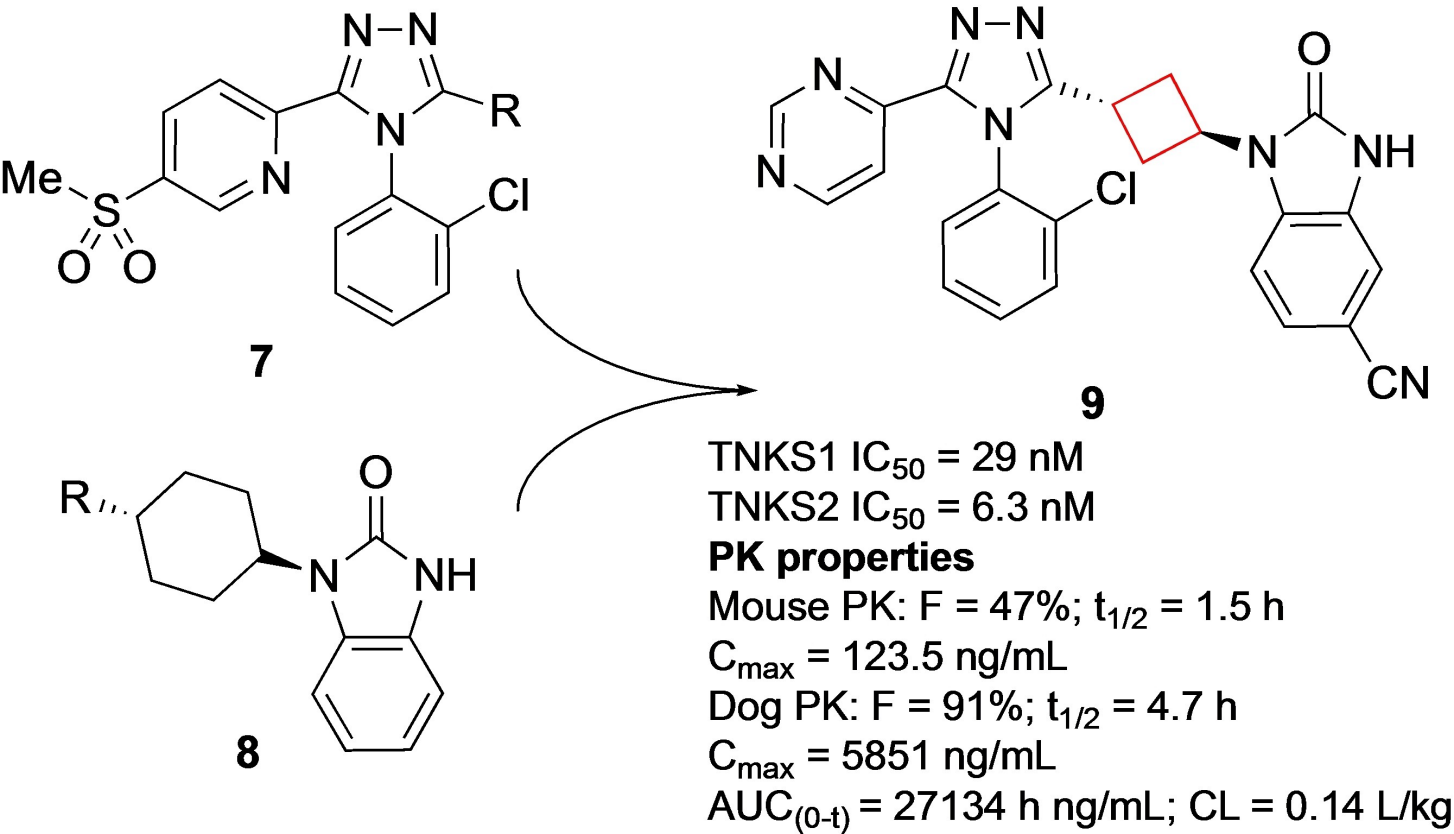
Tankyrase inhibitors **7** and **8**, and optimized inhibitor **9**.

Tankyrase (TNSK) enzymes control several cellular pathways that execute key functions such as mitosis, energy metabolism and cell fate. Inhibition can have therapeutic potential in selected cancers such as colorectal and non‐small cell lung cancers.^[49]^ Anumala et al. previously reported^[50]^ the potent tankyrase inhibitor **7**, which displayed a poor pharmacokinetic (PK) profile. To enhance its PK profile as well as retain its potency, two moieties of known inhibitors were combined using various linkers.^[18]^ Among 1,4‐phenylene, *trans*‐1,4‐cyclohexyl and *trans*‐1,3‐cyclobutyl linkers the latter provided the best balance between rigidity and flexibility. The shorter linker distance also directs the triazole moiety in a slightly different orientation compared to the six‐membered‐ring linkers, allowing the pyrimidine to engage in π‐π interactions more efficiently with a tyrosine residue in the binding pocket. This led also to an improved PK profile (compound **9**; Figure 5).

**Figure 6:**
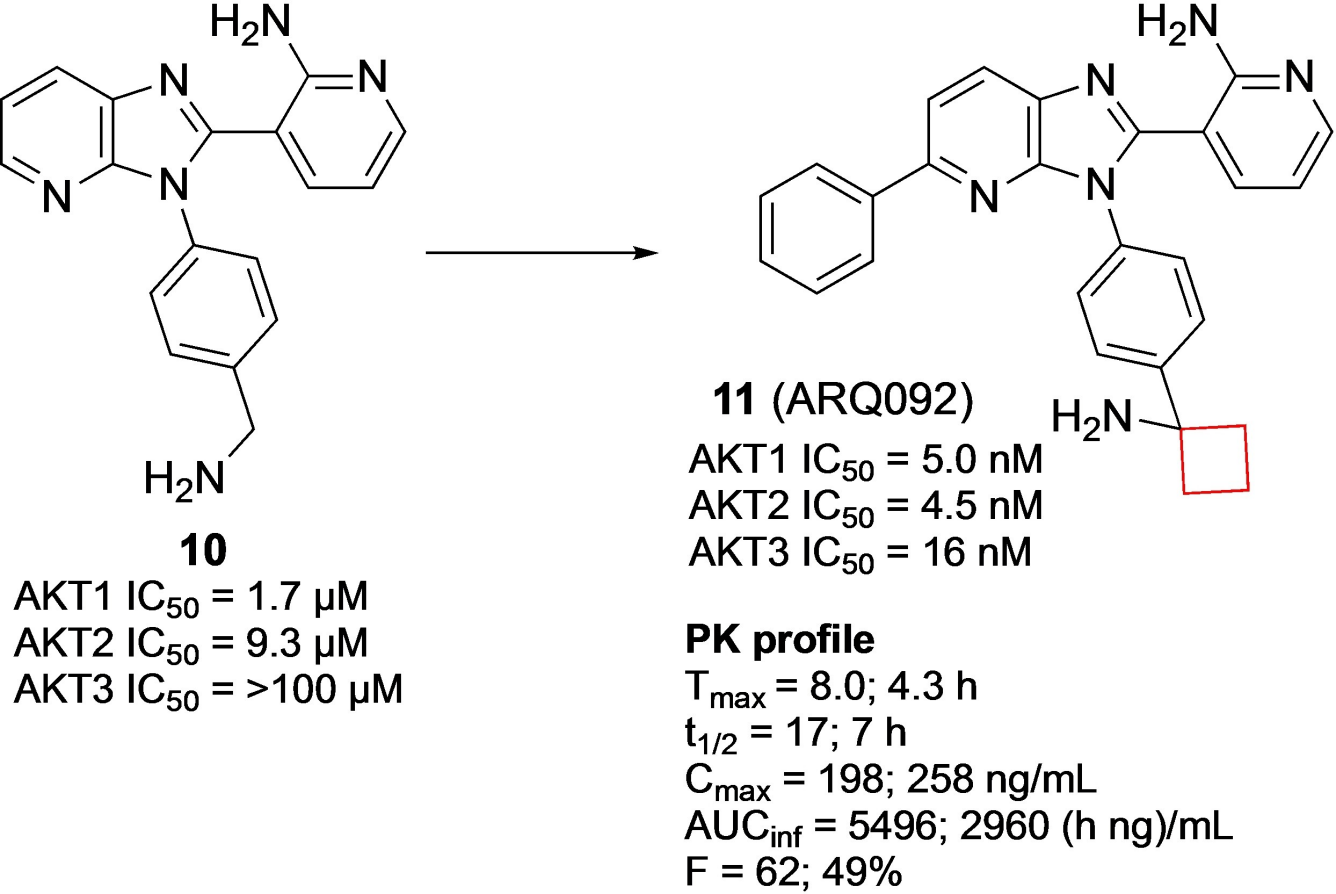
AKT inhibitor **10** and **11**.

Lapierre et al.^[19]^ aimed to develop inhibitors for AKT proteins which are involved in cell proliferation, migration and anti‐apoptotic survival as observed in several human cancers.^[51]^ A previous study indicated that optimizing the activity against AKT1, AKT2, and AKT3 was crucial for achieving biochemical potency and cellular inhibition in multiple tumor types.^[52]^ Though potent, their imidazo[4,5‐*b*]pyridine series, including compound **10**, exhibited low plasma and tumor exposures and therefore the authors aimed to improve the overall PK profile of the AKT inhibitor as well as improve *in vivo* activity. Installation of a cyclobutylamine on the benzylic position resulted in improved inhibition of AKT. The binding mode of this scaffold was investigated by co‐crystallization of **11** (ARQ092; Figure 6) with target AKT1 and revealed its binding mode. The benzylic amine engages in bidentate hydrogen bonds with Tyr272 and Asp274 residues in the binding pocket. This interaction is enabled by the positioning of the cyclobutane in a hydrophobic region of the binding pocket, which resulted in a highly potent analogue. Compound **11** demonstrated high enzymatic potencies against AKT1, AKT2, and AKT3 as well as tumor growth inhibition in human xenograft mouse models of adenocarcinoma and an improved PK profile. It is currently in phase I and II clinical trials for various cancers, Proteus syndrome, and PIK3CA‐related overgrowth spectrum.

**Figure 7:**
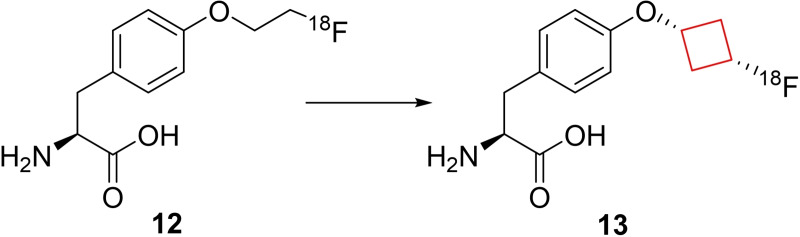
^18^F‐radiolabeled PET tracers **12** and **13**.

In cancer diagnostics, positron emission tomography (PET) is widely applied to locate tumors by visualizing the accumulation of biologically active molecules in tumor tissue. If the radiolabeled molecule is unstable *in vivo*, the visualization tools cannot distinguish between accumulation of the intact molecule or its metabolites. This instability can thus lead to increased background activity and misleading information. In existing therapeutics, often a ^18^F‐fluoroethyl conjugated to a heteroatom is used. These chemical moieties, both unlabeled and radiolabeled were prone to enzymatic degradation.^[53]^ Franck and co‐workers^[35]^ hypothesized that if the ^18^F‐alkyl chain was substituted for a ^18^F‐cycloalkyl group, it would increase the metabolic stability of the PET tracer molecule. The model chosen was a tyrosine‐based amino acid, as *O*‐alkylated tyrosines have been shown to be transported into cancer cells by the large amino acid transporter.^[54]^ The authors replaced the ethyl linker from compound **12** by a *trans*‐cyclobutyl ring (compound **13**; Figure 7). Compound **13** was transported into numerous different cancer cells and despite the bulkier cyclobutyl group the biological properties remained unchanged. *In vitro* stability of the radiolabel in human and rat plasma showed excellent stability displaying over 120 minutes of stability as well as the unlabeled moiety showing over 60 minutes of metabolic stability. This compound is a good starting point for further *in vivo* research and shows that the cyclobutyl linker exhibits improved *in vivo* stability compared to the ethyl‐linked radiolabel and might therefore be a potential candidate as a PET tracer.

**Figure 8:**
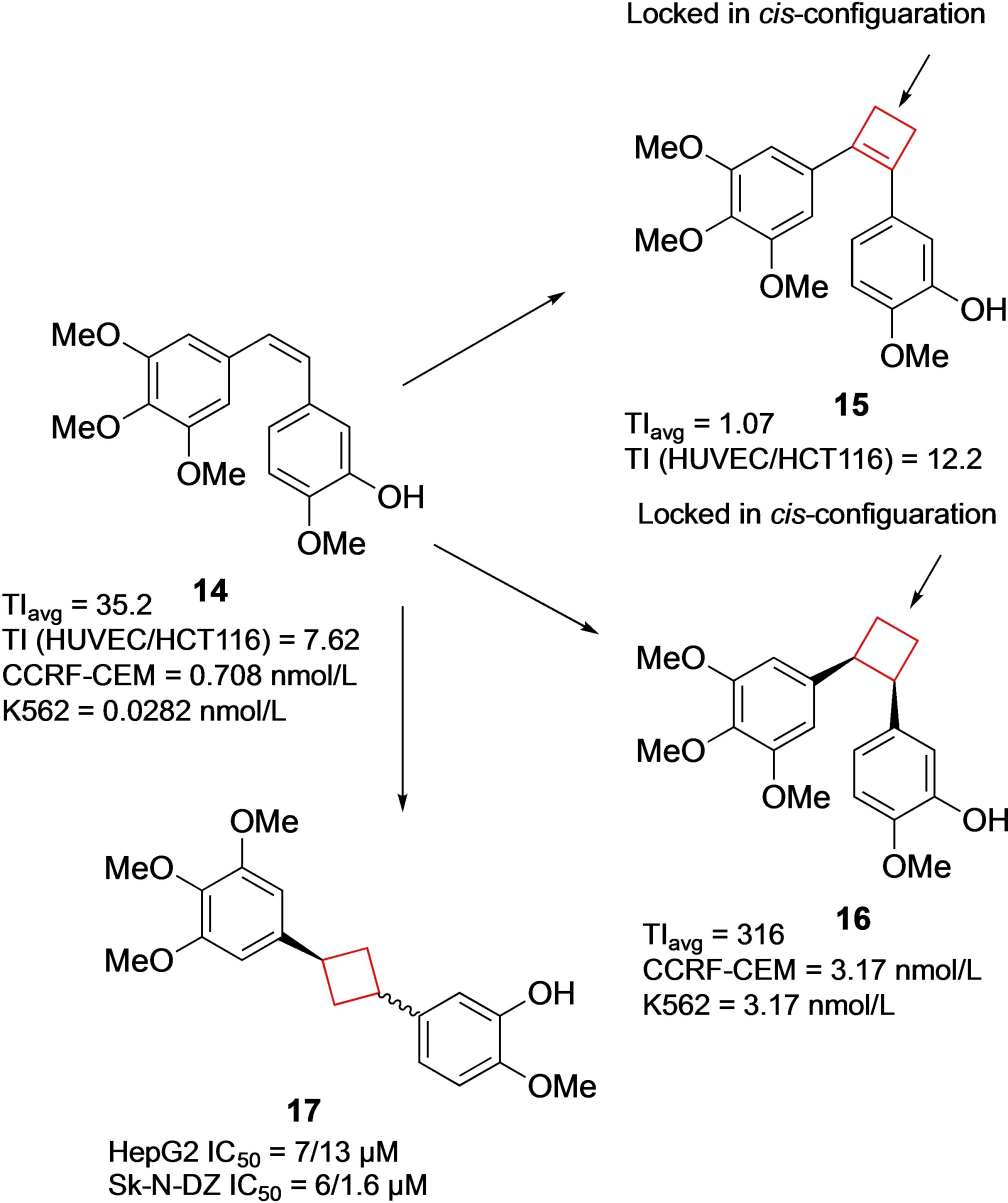
Natural product **14**, its cis‐constrained analogues **15** and **16**, and cis/trans‐1,3‐substituted analogue **17** (data refer to cis/trans isomers, respectively).

Extracts from the South African willow tree *Combretum caffrum* have been used in traditional African medicine.^[55]^ Extensive studies have identified compound **14** as a potent natural drug showing anti‐tumour activity.^[56]^ Its activity is based on inducing apoptosis by selectively binding to the colchicine binding site of tubulin. This inhibits tubulin polymerization which then leads to cell cycle disruption.^[57]^ Several analogues of compound **14** have already been tested in clinical studies. The core structure of these compounds is characterized by a highly oxygenated *cis*‐stilbenoid moiety. The substitution pattern on the aromatic rings was crucial for its activity, as well as the *cis*‐configuration of the alkene in order to efficiently direct the aromatic substituents to the tubulin target. To enhance the *in vivo* potency, Nowikow et al.^[32]^ wanted to overcome alkene isomerization under physiological conditions by chemically locking the scaffold into the *cis*‐orientation. This was achieved by synthesizing saturated and unsaturated *cis*‐constrained carbocycles from cyclobutyl up to cyclohexyl analogues. Biological studies concluded that the larger carbocycle analogues showed lower potencies compared to cyclobutane and cyclobutene analogues. Cyclobutene **15** showed the highest activity towards numerous cancer cell lines, however, with relatively low selectivity. Cyclobutane derivative **16** exhibited comparable potency as the natural product for CCRF‐CEM and K562 cell lines with a high overall therapeutic index (TI) (Figure 8). These compounds therefore form a solid foundation for further *in vivo* evaluations and show how cyclobutanes and/or ‐butenes can be employed to conformationally lock compounds into their most active form.

In a similar study, Malaschuk et al.^[33]^ were following a comparable approach. They synthesized *cis*‐ and *trans*‐1,3‐disubstituted analogues **17** of antitumor natural product **14** to mitigate *in vivo cis*/*trans*‐isomerization (Figure 8). During *in vitro* cytotoxicity evaluations in HepG2 and SK−N‐DZ cell lines it was concluded, however, that their cytotoxicity was rather poor (micromolar range) compared to **15**.

**Figure 9:**
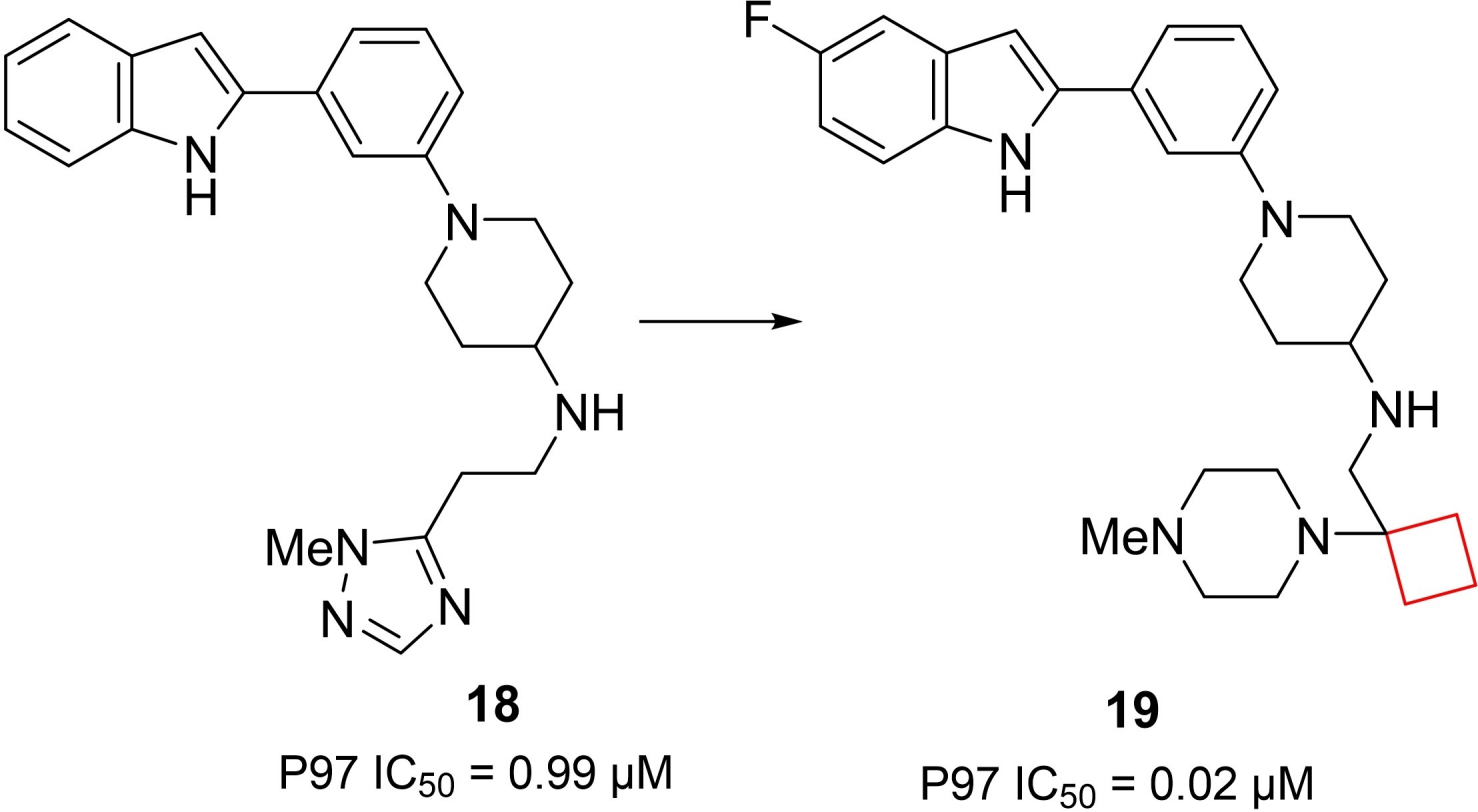
P97 inhibitors, optimized HTS hit **18** and optimized lead **19**.

The p97 ATPase protein plays a key role in protein homeostasis. This protein facilitates degradation of polypeptides by the proteasome with energy supplied from the hydrolysis of ATP. Clinical success of other proteasome inhibitors in certain cancers suggests that p97 inhibitors may potentially become anticancer drugs.^[58]^ In an effort to develop novel allosteric p97 inhibitors, Laporte and co‐workers^[24]^ identified a 2‐phenylindole scaffold in a HTS screen. The indole moiety appeared crucial in structure activity relationship (SAR) studies, leading to optimized compound **18** (Figure 9). To further improve its activity, a SAR study was conducted on the side chain keeping the 2‐[3‐(piperidin‐1‐yl)phenyl]‐1*H*‐indole constant. The linker length did not show a significant effect, but replacement of the triazole by a piperazine as the terminal substituent yielded a 3‐fold increase in potency. The authors then chose to conformationally restrict the ethyl linker and opted to introduce a cyclobutyl ring after other restriction methods were unsuccessful. Interestingly, the cyclobutyl did not only conformationally restrict the scaffold, but also introduced a kink into the flexible linker. This structural change gave a remarkable 10‐time increase in potency compared to **18**. This unexpectedly high potency was hypothesized to be driven by desolvation of the cyclobutyl group in a more buried binding mode. These compounds now provide a good starting point for further in‐depth studies and have the potential to be developed into a novel class of cancer therapeutics.

**Figure 10:**
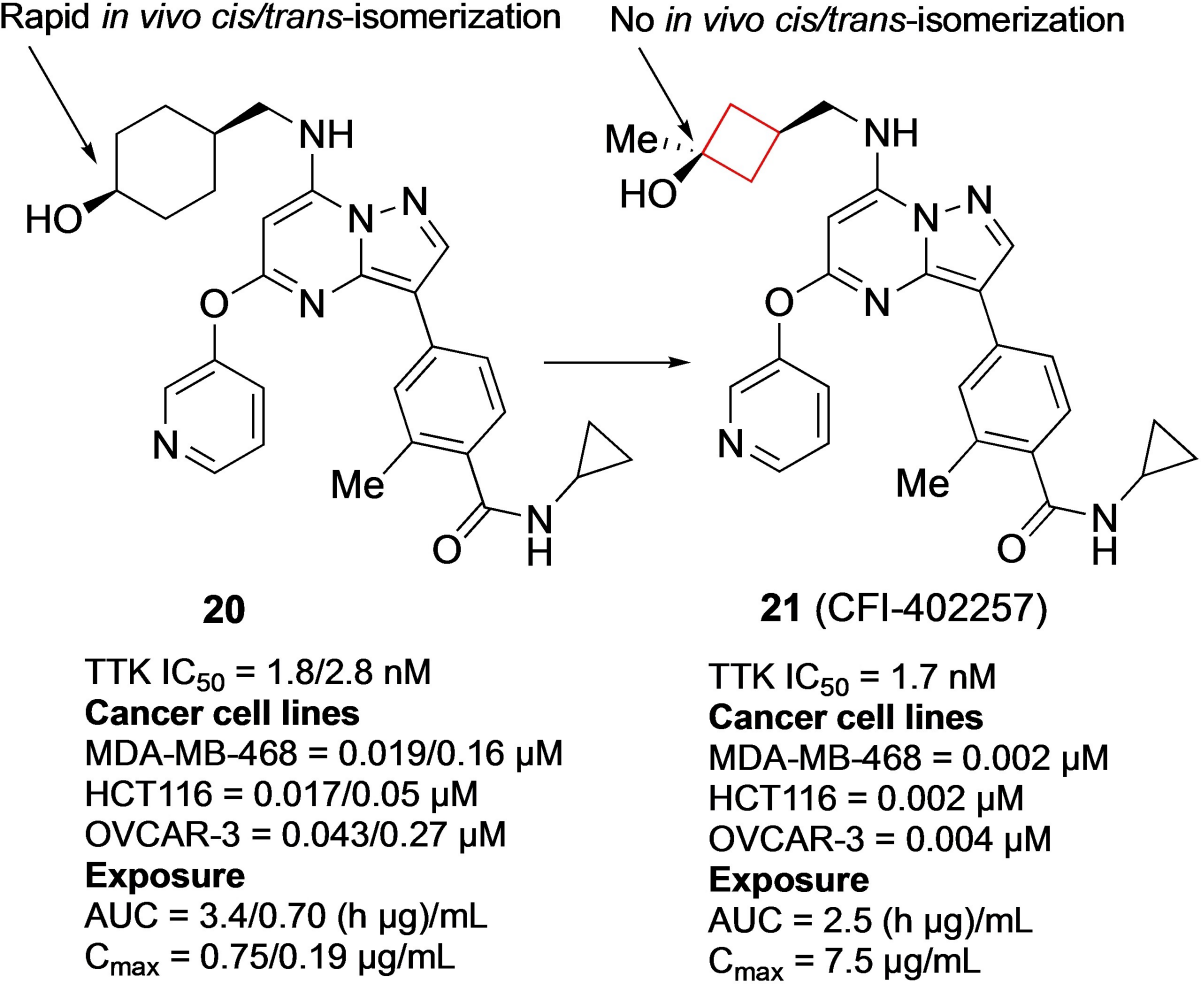
TTk inhibitors **20** (values refer to cis/trans isomers, respectively) and **21**.

Threonine tyrosine kinases (TTK) are often overexpressed in breast cancer cells.^[59]^ Patients displaying high TTK levels generally have high tumor grades, usually resulting in poor clinical outcomes.^[60]^ Inspired by a carbon‐to‐nitrogen switch from imidazo[1,2‐*a*]pyridazines to imidazo[1,2‐*b*]pyridazines in patent literature, Liu and co‐workers^[31]^ reasoned a similar transformation for pyrazolo[1,5‐*a*][1,3,5]triazines would result in a more optimal PK profile as TTK inhibitors. During the SAR study, the authors aimed to modulate the physiochemical properties while maintaining *in vitro* potency and bioavailability. This goal was pursued by installing a hydroxy group on the solvent exposed region in the binding pocket and adding a weakly basic group to the aromatic hydrophobic core. By installing a cyclohexanol ring on this region it appeared that they were potent TTK inhibitors, but that only the *cis*‐isomer **20** (Figure 10) exhibited desirable oral exposure and cell activity. Unfortunately, *in vivo* this stereoisomer was rapidly converted into the *trans*‐isomer. The authors installed a cyclobutanol instead of a cyclohexanol that exhibited no *in vivo* isomerization, but at the cost of oral exposure. This analogue was modified to increase the steric bulk by the incorporation of a methyl group to obtain **21** (CFI‐402257) (Figure 10). This *cis*‐cyclobutanol analogue is a potent TTK inhibitor and exhibited the highest bioavailability in mice. This compound displays the use of the cyclobutyl ring for rigidity increase when stereochemical interactions are a major contributing factor towards oral exposures and activities. In addition, this analogue also exhibited TTK kinase selectivity over 262 other kinases and is currently in phase I and II clinical trials for breast and advanced solid cancers.

**Figure 11:**
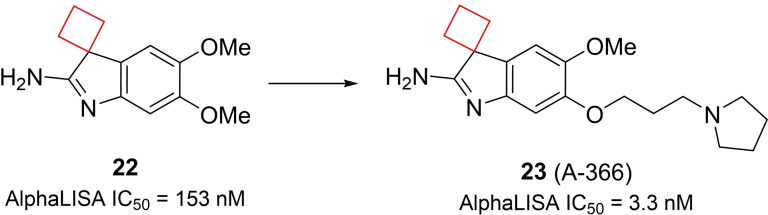
G9a inhibitors **22** and **23**.

Histone methyltransferases (HMTs) are epigenetic enzymes catalyzing methylation of histone lysines and arginines. They have emerged as therapeutic targets as this process modulates transcription^[61]^ and its dysregulation is implicated in numerous forms of cancer.^[62]^ Euchromatic histone methyltransferase 2, otherwise known as G9a, is an example of such an HMT which has been linked to various cancers.^[63]^ Sweis et al.^[64]^ searched for chemically distinct G9a inhibitors through screening of an in‐house compound library. Compound **22** stood out having submicromolar potency (IC_50_=153 nm) and contained a spirocyclic cyclobutane ring that in SAR studies was found to be crucial for its potency towards G9a. Modification to a spirocyclic cyclopentane, cyclohexane or substitution to hydrogens resulted in potency drops of at least one order of magnitude. Spiro[cyclobutane‐1,3′‐indol]‐2′‐amine **23** (A‐366) (Figure 11) was found optimal during SAR studies and the authors conducted an X‐ray cocrystal study. Interestingly, the cyclobutyl moiety was shown to reside close to the polar Asp1078 residue, which could not be explained by the authors. This result nevertheless provided a potent inhibitor which was found to be selective over 21 other HMTs.

**Figure 12:**
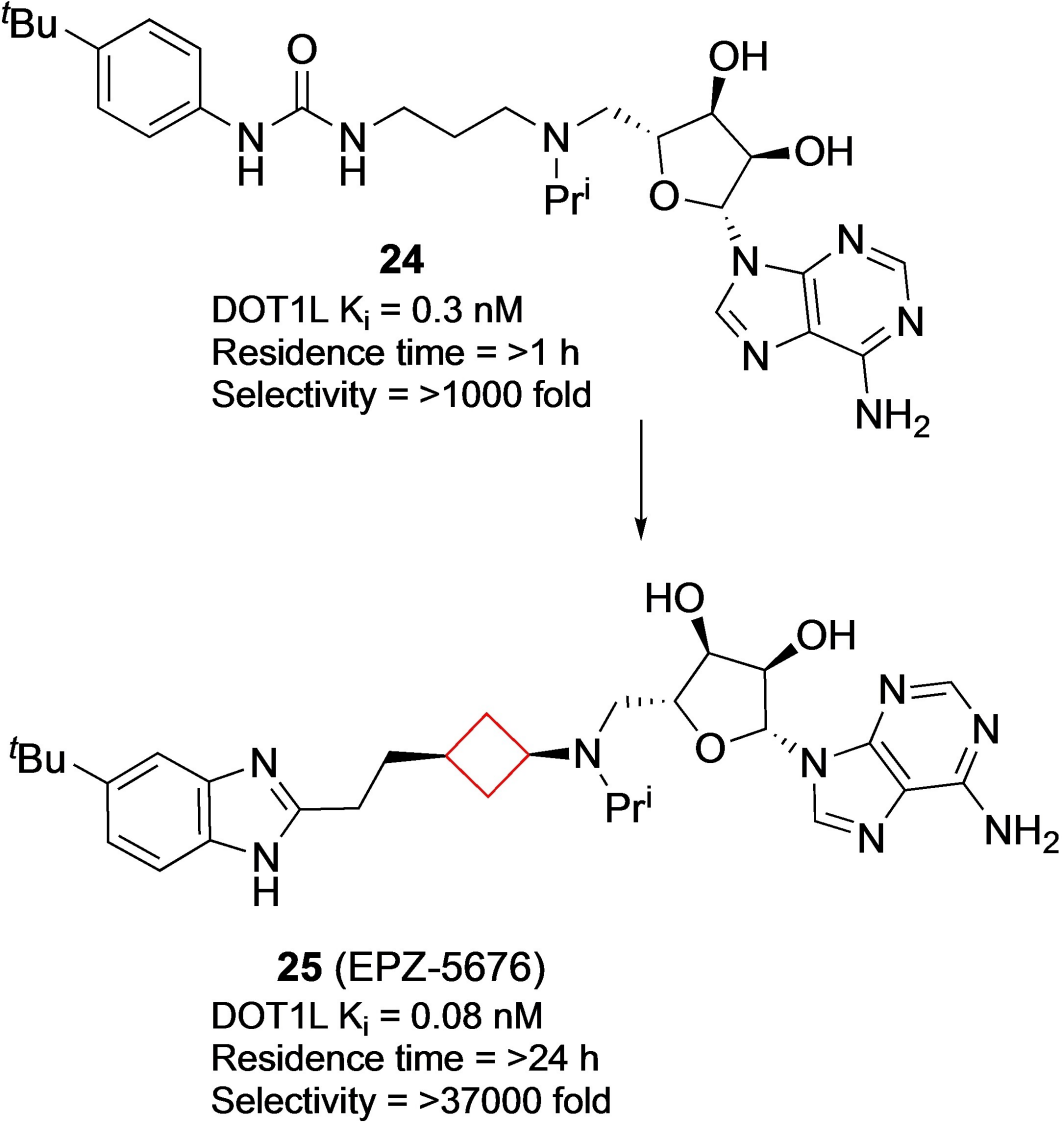
DOT1L inhibitors **24** and **25**.

Daigle and co‐workers^[65]^ aimed to identify small‐molecule inhibitors for H3K79 HMT protein DOT1L. This enzyme is involved in processes such as DNA‐damage response, gene expression and cell cycle progression and has also been implicated in the development of mixed lineage leukaemia (MLL).^[66]^ People with MLL often have a methylation of H3K79 that should not occur and therefore inhibiting the methylation might prevent the formation of MLL.^[67]^ Compound **25** (EPZ‐5676, Figure 12) was identified as a potent small‐molecule inhibitor of DOT1L based on a structure‐guided design and optimization starting from known aminonucleoside inhibitor **24**, whose pharmacokinetic profile was not optimal and was rendered unsuitable for clinical development. Even though the chemical rationale behind the transformation towards **25** was not published, it exhibited a strong potency towards DOT1L, inducing conformational changes as well as showing high selectivity over other protein methyltransferases, improved residence time and *in vivo* efficacy. This compound was submitted to clinical trials and is currently ongoing in phase I and II trials for treatment of leukaemia.

### 2.2 Cyclobutanes in autoimmune disease research

**Figure 13:**
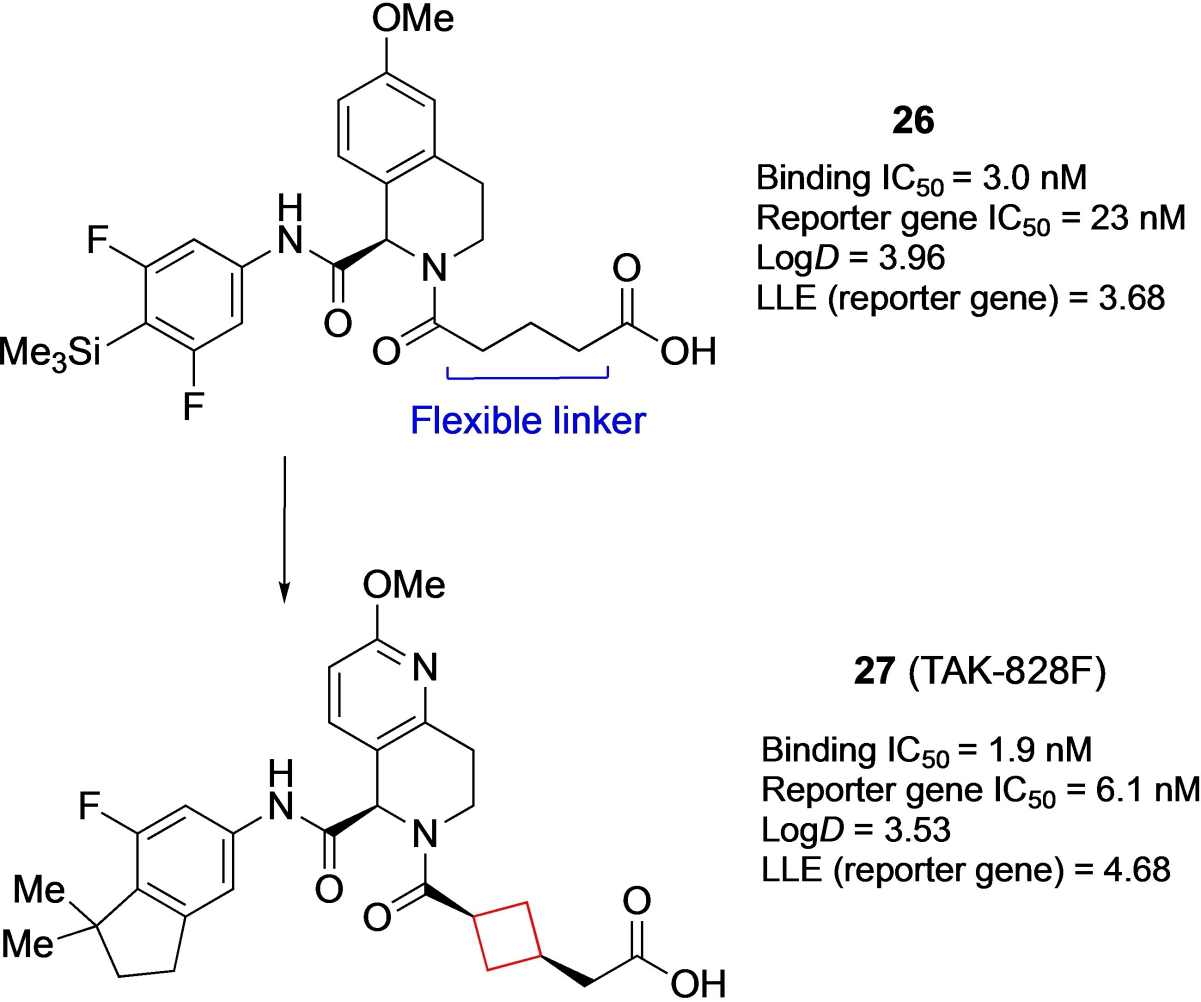
RORγt inhibitors **26** and **27**.

Compound **27** (TAK‐828F, Figure 13 *)* is a nuclear receptor retinoic acid receptor‐related orphan receptor gamma (RORγt) inhibitor. This protein plays an important role in regulating the immune response in human TH17 cells[^68^, ^69^] and has been suggested to play a pathogenesis role in autoimmune diseases such as rheumatoid arthritis and psoriasis.^[70]^ RORγt plays a crucial role in the function and differentiation of TH17 cells^[69]^ and modulators thereof can be regarded as potential candidates for treatment of immunological diseases. Previously, Kono et al. had already reported on the favorable *in vitro* RORγt and PK properties of **26**.^[71]^ The aim was to retain or improve the attractive biological profile as well as lower its lipophilicity, since it was non‐optimal for drug delivery. The highly lipophilic trimethylsilyl group was swapped and cyclized to an indane ring. In addition, the tetrahydroquinolone was altered to a pyridine, with the addition of a methoxy substituent as well. These alterations reduced its lipophilicity substantially (log *D=*3.96 originally vs 3.39 after modification) and retained its *in vitro* potency. Lastly, the authors hypothesized that a more rigid linker would be beneficial as the entropy loss upon binding would be minimal. Multiple cyclobutane linked compounds were synthesized; the *cis*‐1,4‐cyclobutane linker yielding compound **27** was optimal upon *in vitro* testing, while the lipophilicity was only slightly increased compared to the flexible linker. Compound **27** also showed the highest plasma exposure and oral bioavailability in *in vivo* studies in mice at 1 mg/kg.^[38]^ This compound has undergone phase I clinical studies with no follows up as of now.

**Figure 14:**
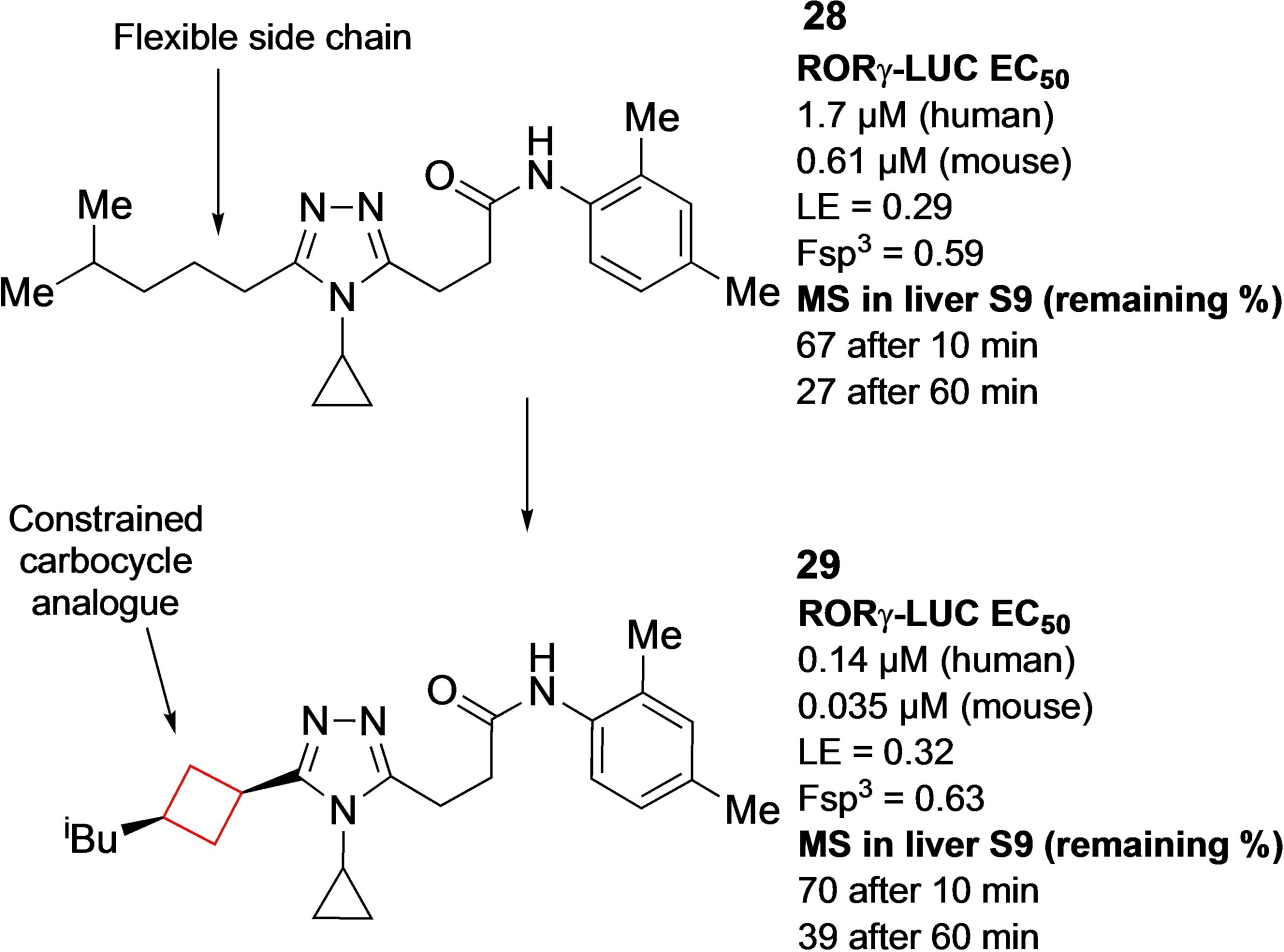
RORγt inhibitors optimized HTS hit **28** and optimized lead compound **29**.

Hirata and co‐workers^[39]^ sought to identify novel RORγt inhibitors by means of a HTS. The initial screening hit showed a structurally unique molecule with moderate potency against RORγt (hLUC EC_50_=1.7 μM, FRET EC_50_=0.85 μM) with >20‐fold selectivity over five other nuclear receptors. This compound had several drawbacks such as modest time‐dependent human cytochrome (CYP) 3A4 inhibition and low liver microsomal stability. The authors hypothesized that by improving the ligand efficiency (LE) from 0.25 to at least 0.30,^[72]^ and decreasing the lipophilicity would yield an improved drug profile. An additional strategy employed by the authors was to increase its fraction of saturated carbons (Fsp^3^), as a decrease in Fsp^3^ would result in an increased incidence of CYP inhibition.^[73]^ After initial SAR studies and X‐ray co‐crystal analysis of intermediate **28** (Figure 14) the conclusion was made that the inhibitor was bound in a U‐shaped conformation and that van der Waals contact was important for its binding efficiency. Therefore, the authors modified the side chain of the 1,2,4‐triazole moiety by incorporating cyclobutane residues to stabilize their binding conformation and mask potential metabolic sites. These analogues exhibited the best LE values with slight increases in metabolic stability as well as their Fsp^3^ values with *cis*‐cyclobutane analogue **29** (Figure 14) being optimal. This derivative was subjected to *in vivo* experiments showing promising results that confirmed the authors original hypothesis.

**Figure 15:**
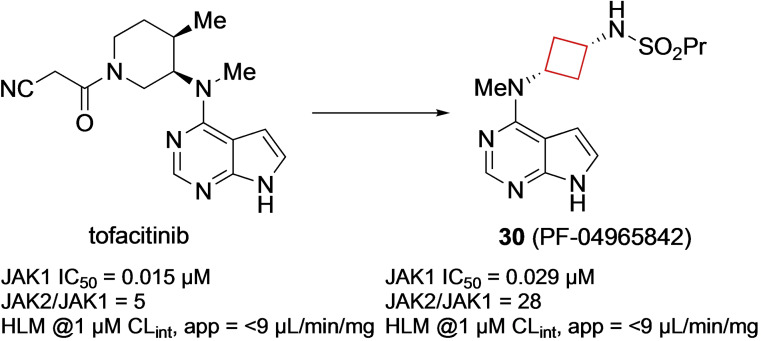
Tofacitinib and JAK1 inhibitor **30**.

Compound **30** (PF‐04965842) is a selective intracellular Janus kinase 1 (JAK1) inhibitor. The JAK family of enzymes are central to inflammation and regulation of immunity.^[74]^ This property makes them attractive drug targets for autoimmune diseases.^[75]^ Based on the pre‐existing drug tofacitinib, Vazquez et al.^[20]^ kept the pyrrolopyrimidine moiety, being central to the hinge binding, subsequently evaluating a range of diamine linkers. The result being that cis‐1,3‐cyclobutane diamine linkers tended to exhibit not only potencies in the low nanomolar range but also excellent selectivity in the JAK family of enzymes, as shown in Figure 15. The authors subsequently investigated the relation the cyclobutyl moiety had on the binding interaction within the JAK family. The puckered conformation of the cyclobutyl ring allowed for the sulphonamide NH to be in a position to form hydrogen bonds with Arg and Asn residues of JAK1. The trans‐isomer of **30** showed less favourable activities because this interaction cannot be fully achieved. Because the favourable activity profile, this compound is currently in phase III clinical trials for treatment of atopic dermatitis.

### 2.3 Cyclobutanes in CNS disease treatment

**Figure 16:**
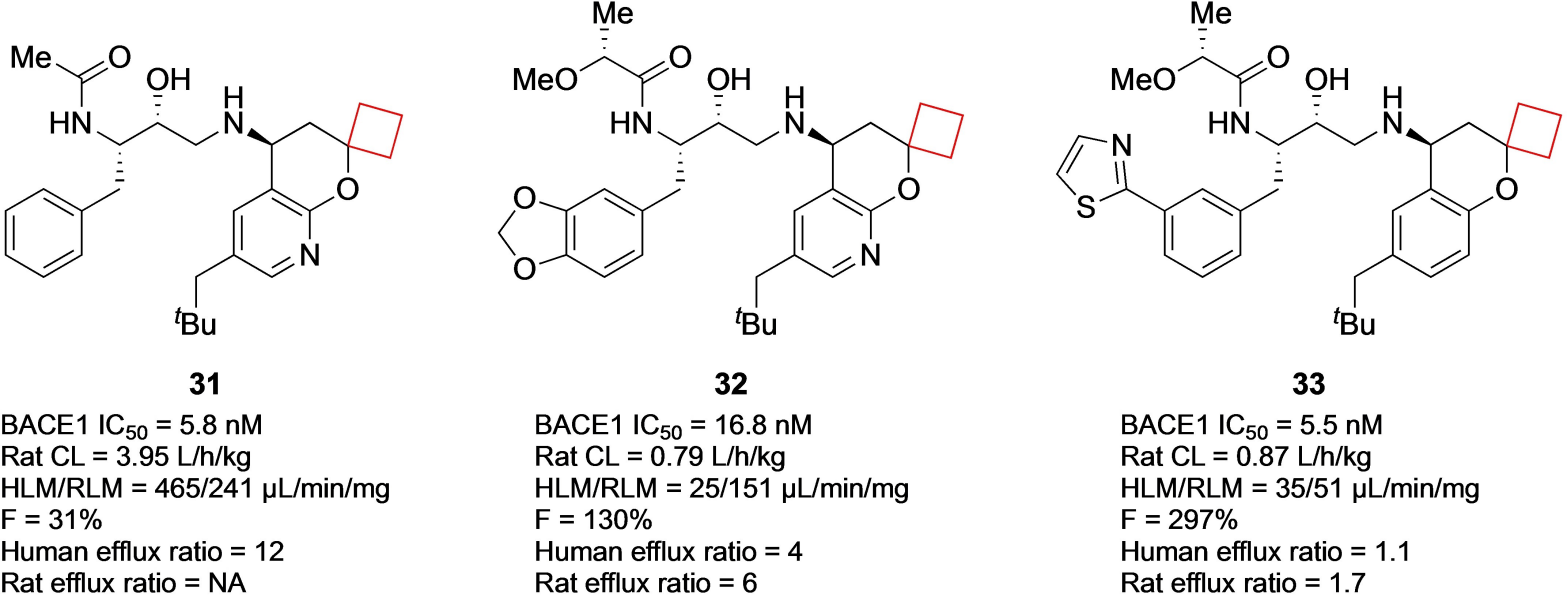
BACE1 inhibitors **31**, **32**, and **33**.

Weiss and co‐workers^[25]^ set out to identify potent β‐site amyloid precursor protein cleaving enzyme 1 (BACE1) inhibitors from a known set of inhibitors, aiming to optimize their suboptimal pharmacokinetics and central nervous system (CNS) partitioning. The BACE1 protein is considered as a therapeutic target for the treatment of Alzheimer's disease (AD), as it is involved in the formation of Aβ peptides. Previously reported hit **31** ^[76]^ (Figure 16) showed low nanomolar potency against BACE1 in addition to reducing Aβ_40_ levels. This was further optimized by SAR studies to compound **32**, having a better overall PK profile with lower rat clearance (CL) and metabolic stability in the form of human and rat liver CL_int_, at the overall loss of some potency. This compound was co‐crystallized with BACE1 peptide to find outs its binding mode. The benzodioxolane ring system inhabits the S1 binding pocket. The spirocyclic cyclobutyl ring and the neopentyl group occupy the S1′ and the S2′ hydrophobic pockets respectively, resulting in a potent binding efficiency. Later efforts^[26]^ further optimized this moiety based on its intrinsic clearance and CNS penetration resulting in **33**.

**Figure 17:**
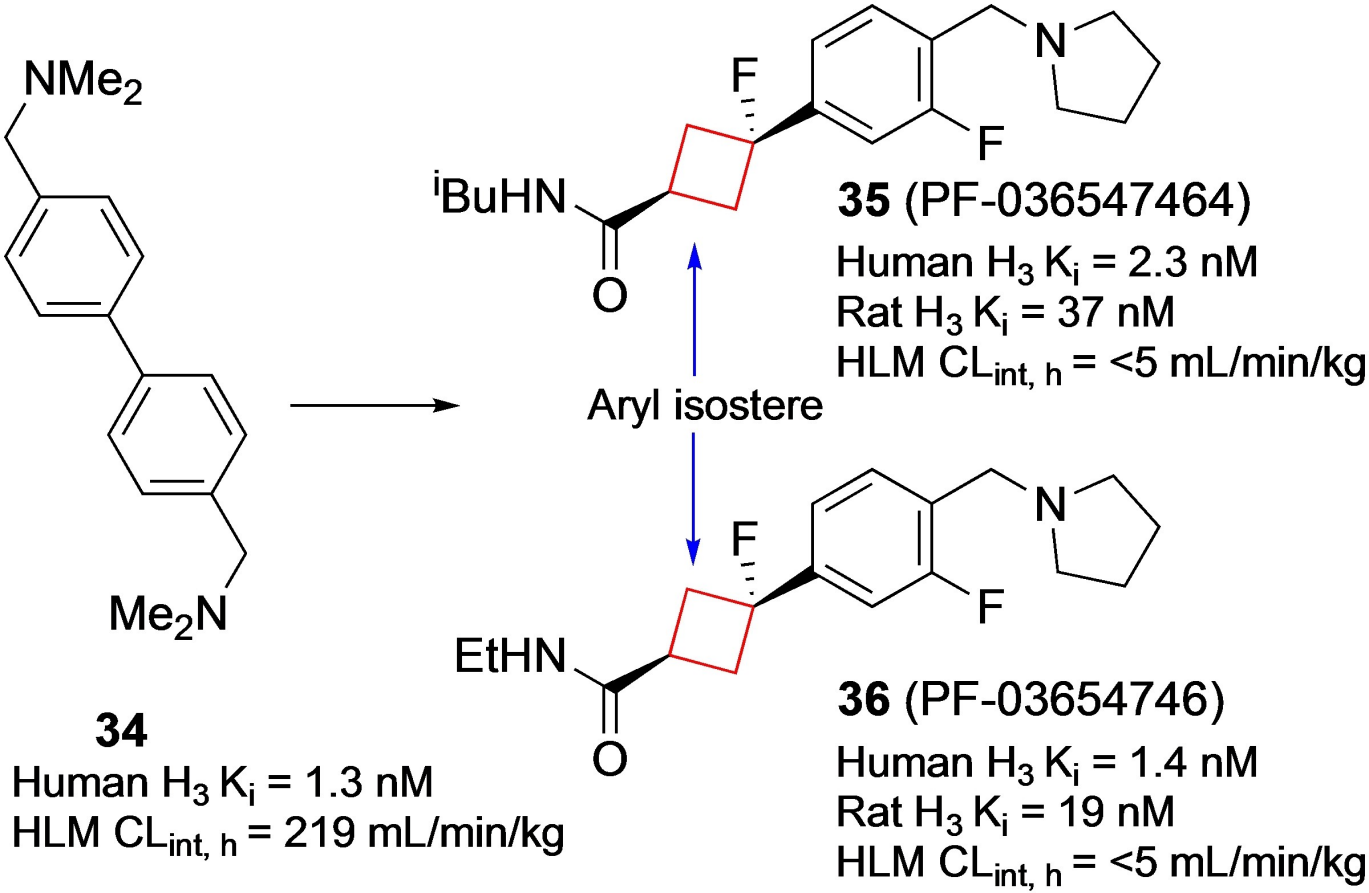
H_3_R inhibitors, initial HTS hit **34** and optimized compounds **35** and **36**.

Researchers from Pfizer^[37]^ discovered two clinical candidates **35** (PF‐03654746) and **36** (PF‐03654764) as histamine 3 receptor (H_3_R) antagonists. Though numerous H_1_ and H_2_ receptor antagonist pharmaceuticals have been developed, H_3_ and H_4_ receptor antagonists are overall less developed.^[77]^ H_3_ receptors have their highest densities in the prefrontal regions of the brain, where they play a central role in numerous functions including learning, arousal and wakefulness.^[78]^ It is suggested that pharmaceutical antagonists of H_3_ receptors can be therapeutic agents for attention deficit hyperactivity disorder (ADHD), AD, narcolepsy and other cognitive disorders.^[79]^ Pharmaceuticals for these conditions are generally effective, but can also induce (toxic) adverse effects. The authors therefore sought to develop safer and more selective H_3_ antagonists for CNS disorders. An early HTS identified compound **34** (Figure 17) and exhibited high affinity for human H_3_ receptors (K_i_=1.3 nM) but also a significant estimated unbound CL_int_ (CL_int, u_=219 mL/min/kg). In addition, it was suspected that the biaryldiamine moiety might induce safety issues as similar structures are known to be genotoxic.^[80]^ The authors searched for aryl isosteres to mimic the distance between the two basic groups and theorized that a cyclobutane linker might act as one. This hypothesis was confirmed by molecular modelling studies, where the two structures exhibited good overlap of the two basic groups. Furthermore, the authors theorized that the 3D volume of the cyclobutane ring would be less likely to induce DNA damage by binding in the minor groove of DNA. Therefore, one aryl group was substituted for a *trans*‐cyclobutyl ring. After numerous further optimization studies mostly regarding the PK and safety profiles, structures **35** and **36** identified as optimal compounds.

These leads displayed good absorption, distribution, metabolism and excretion (ADME) properties and displayed negative *in vitro* micronucleus assay results and no *in vivo* phospholipidosis (PL), thus displaying favorable safety properties. A handful of phase I and II trials were conducted for both leads, with no continuation to phase III as of yet.

**Figure 18:**
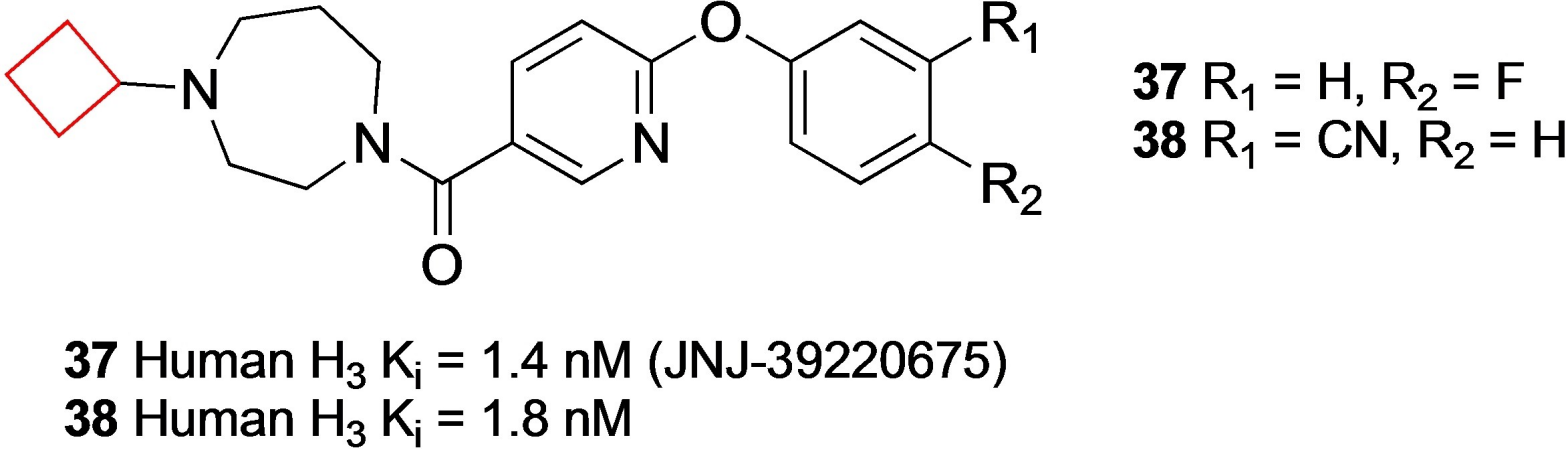
H_3_ antagonists **37** and **38**.

Letavic et al.^[81]^ also searched for novel preclinical H_3_ receptor antagonists to develop into clinical candidates. An initial aryl‐oxynicotinamide showing moderate affinity for the H_3_ receptor and no affinity for SERT inspired the authors to expand this series. SAR studies showed that a diazepane ring was preferred over a piperazine ring, with cyclic substituents on the diazepane moiety. Cyclobutyl derivatives **37** and **38** (Figure 18) exhibited an optimal balance between microsomal stability, rat plasma and brain concentrations and receptor occupancy. During further profiling studies, these compounds exhibited H_3_ occupancy at low plasma concentrations, promoted wake and increased histamine release in rat studies in combination with a favorable PK profile. Compound **37** (JNJ‐39220675) has been submitted to clinical trials, but there was no continuation after phase II studies.

**Figure 19:**
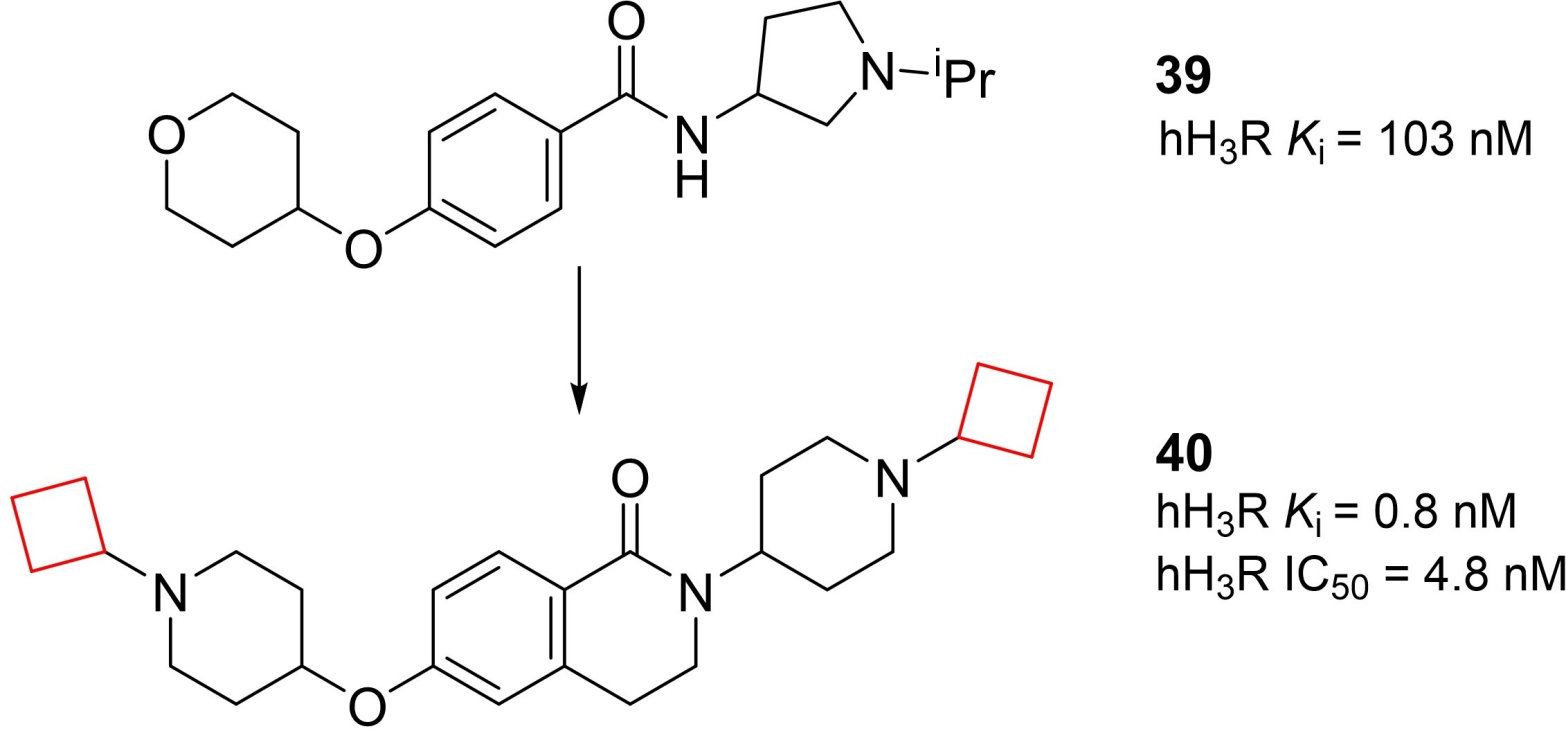
H_3_ antagonists **39** and **40**.

Nirogi and co‐workers^[82]^ aimed to identify novel H_3_ receptor antagonists as well. Based on the challenges with H_3_ receptor antagonists such as CYP inhibition, human ether‐a‐go‐go‐related gene (hERG) and PL,^[83]^ the authors aimed to identify compounds that exhibited promising profiles regarding the earlier described properties while also maintaining *in vivo* activity. They identified a series of benzamide compounds with moderate affinities by means of an initial screening and set out to optimize these series. First, cyclization of the amide nitrogen yielded a more optimal balance of rodent half‐life and brain penetration. Numerous *N*‐alkyl substituents on both piperidine rings were investigated to test their activity. It was concluded that cyclic substituents on these positions were well tolerated though the most optimal combination was compound **40** (Figure 19) bearing a *N*‐cyclobutyl substituent on both piperidine rings. This compound was subjected to further *in vitro* testing and appeared both metabolically stable and exhibiting minimal hERG inhibition.

**Figure 20:**
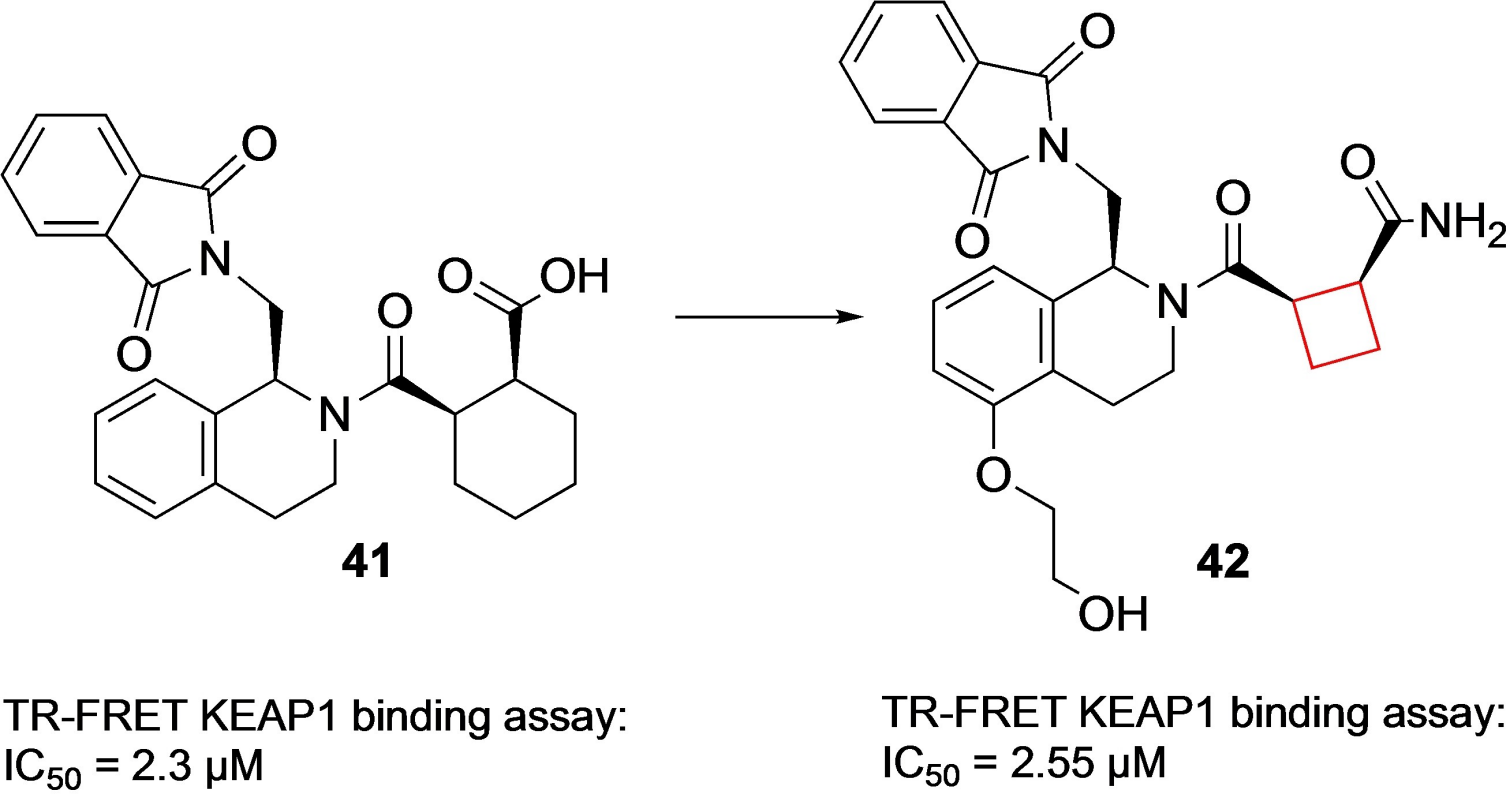
KEAP1 inhibitors **41** and **42**.

Ontoria et al.^[21]^ looked into novel small‐molecule inhibitors of the kelch‐like ECH associated protein 1 (KEAP1)/nuclear factor erythroid derived 2 (NRF2) interaction. This protein‐protein interaction is a key interaction in the NRF2‐antioxidant responsive element mechanism of defence against oxidative stress. Inhibiting the KEAP1/NRF2 interaction, and hence proteasomal degradation, promotes the intracellular concentration of NRF2.^[84]^ This increase plays a key role in anti‐oxidant processes to prevent neurodegenerative diseases such as Huntington's and Parkinson's.^[85]^ Previous research^[86]^ identified an oligopeptide as the minimal binding sequence to the Kelch domain of KEAP1. A number of crystal structures of KEAP1 cocrystallized with this oligopeptide provided crucial structural information regarding the binding interactions with the protein. Based on this information, small‐molecule inhibitor tetrahydroisoquinoline **41** (Figure 20) was designed. This inhibitor however, which was shown to occupy only 3 of the 5 available binding sites, lacked binding affinity compared to small‐molecule inhibitors binding to all 5 available sites. In addition, **41** contains a carboxylic acid group and although favorable in this interaction, in general acidic moieties are avoided in CNS active pharmaceuticals as they commonly display poor blood‐brain barrier (BBB) penetration. The authors conducted a small SAR study on the glutamic acid position of the previously identified oligopeptide to gain more insight into the binding mode of the KEAP1 binding site. It was found that peptide derivatives showed a preference for non‐natural cyclobutylaniline (Cba) residues, where the ring expanded or ring‐contracted analogues both showed sharp decreases in affinity. In addition, a preference for carboxamides was found in this study. When docking the Cba peptide analogue and comparing it to **41**, it was revealed that the cyclobutyl and the cyclohexyl occupied the same region of KEAP1. The cyclobutyl ring in the oligopeptide appeared to direct the primary amine in a favorable position in the binding pocket. Based on these findings, the authors constructed carboxamide‐substituted cyclobutyl derivatives of **41**. The direct comparison of **41** with its ring‐contracted cyclobutyl carboxylic acid analogue resulted in a lower binding affinity due to reduced van der Waals contact. Its analogue though, both carboxamide ring‐contracted as well as hydroxyl substituted, resulted in similar binding affinities compared to **41**. The authors speculated an alternative binding mode with KEAP1 was prevalent compared to **41**. Therefore, X‐ray crystallography studies of **42** (Figure 20) with KEAP1 were performed which indeed resulted in an altered binding mode. The amide of the cyclobutyl derivative is directed in such a way it can form three different direct hydrogen bond interactions with Ser363, Asn414 and Arg415 in KEAP1’s P2 binding pocket. This compound now presents a more robust CNS drug candidate compared to previously identified acidic KEAP1 pharmaceuticals due to the projected BBB penetration.

**Figure 21:**
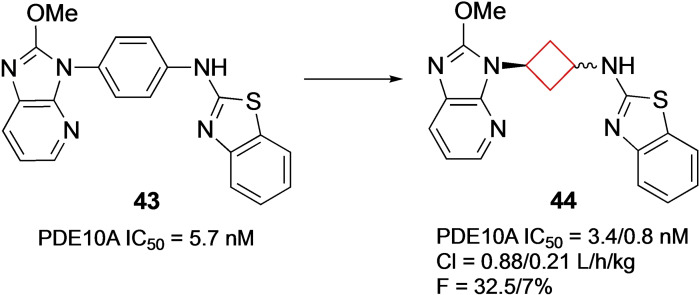
PDEA10 inhibitors **43** and **44** (values refer to cis/trans isomers, respectively).

Phosphodiesterase (PDE) enzymes are involved in intracellular signalling of cyclic adenosine monophosphate (cAMP) and cyclic guanosine monophosphate (cGMP) by converting them to AMP and GMP. Their isoforms are highly localized with 10 out of 11 localized in the CNS. Therefore, controlling their activity in the CNS could potentially be beneficial towards neurodegenerative and neuropsychiatric conditions such as Alzheimer's and Parkinson's disease.[^87^, ^88^] Hu and co‐workers^[22]^ tried to identify PDE10A inhibitors, as its inhibition could present a novel target for the treatment of schizophrenia.^[87]^ This enzyme is highly expressed in brain tissue, especially in the striatum, thought to be disregulated in schizophrenia and an antagonist may have therapeutic benefit. The authors previously identified a PDE10A inhibitor that exhibited low nanomolar potencies (IC_50_=4.5 nM) with moderate rat clearances (Cl=0.53 L/h/kg) and a low bioavailability (F=10 %).^[89]^ Focussing on reducing its metabolic liability, initial SAR studies led to compound **43** (Figure 21). After an X‐ray co‐crystal analysis with human PDEA10 it was concluded that the phenyl linker made no apparent interactions within the binding pocket, thereby posing an opportunity to introduce new interactions, while maintaining the linear orientation between the imidazo[4,5‐*b*]pyridine and the benzo[*d*]thiazol‐2‐amine units. After cyclohexyl linkers showed a reduction in potency, cyclobutyl derivatives were synthesized. Both *cis*‐ and *trans*‐linked cyclobutyl analogues **44** showed low nanomolar potency (Figure 21). Both isomers were used in X‐ray cocrystal structure studies in human PDE10A. While the cyclohexyl analogue was hypothesized to be too bulky to fit in the channel between Gln716 and Tyr683 residues in the binding pocket, the cyclobutyl derivatives fitted well and allowed imidazo[4,5‐*b*]pyridine and the benzo[*d*]thiazol‐2‐amine moieties to engage in critical hydrogen bonding interactions within the binding pocket. The *cis*‐isomer showed more potent binding, but its bioavailability was significantly lower, while the cyclobutyl series generally exhibited a satisfactory PK profile

**Figure 22:**
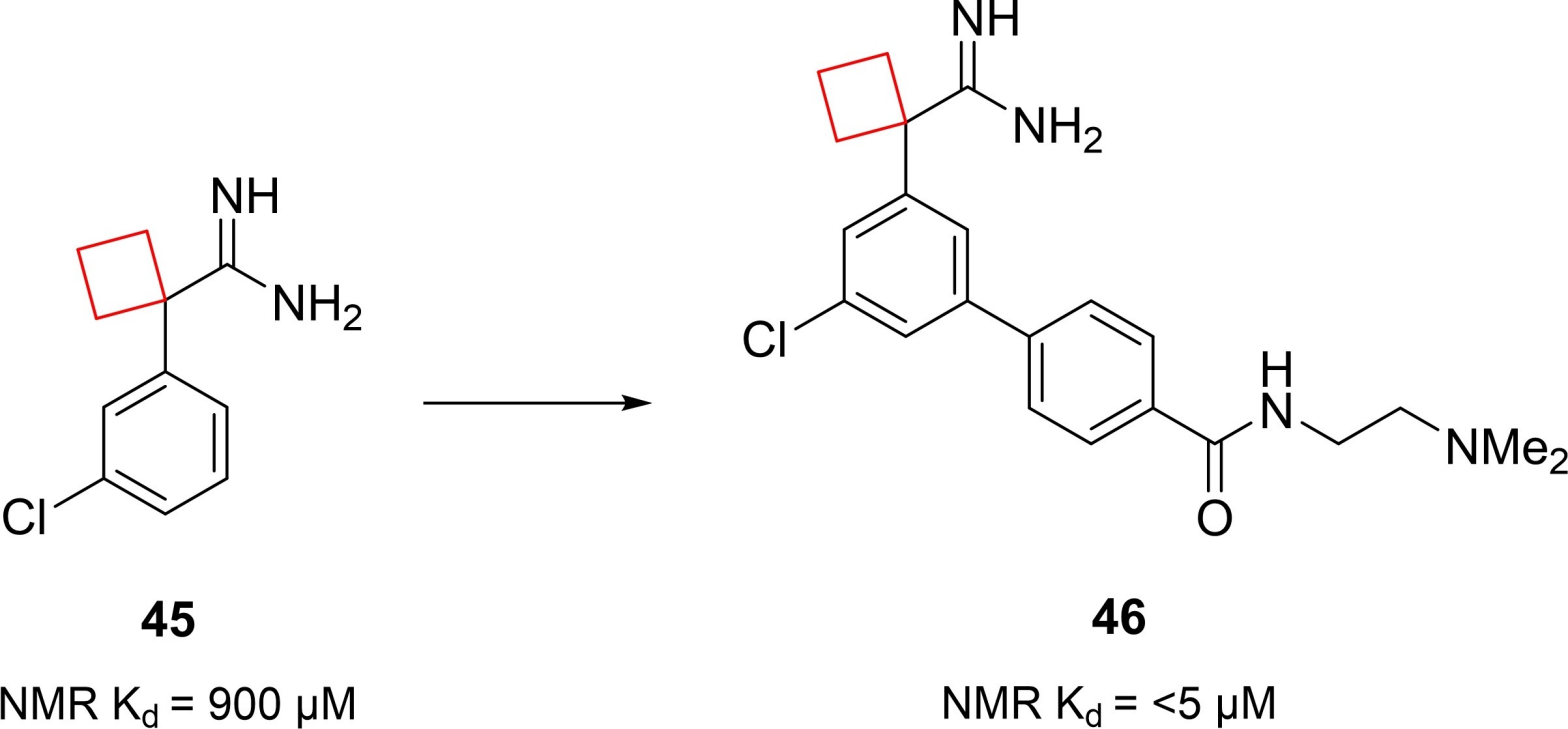
ApoE4 stabilizers **45** and **46**.

Apoliprotein E (apoE) is a lipid carrier protein playing a major role in lipid homeostasis mostly manufactured in brain and liver tissues.^[90]^ This protein has three isoforms namely apoE2, apoE3 and apoE4. The possession of apoE4 alleles have been shown to be a strong genetic risk factor for late‐onset Alzheimer's disease (LOAD).^[91]^ The exact role of apoE4 is unknown,^[92]^ but it was hypothesized that stabilizing the protein may impact its disease pathology. Petros et al.^[27]^ aimed to identify such stabilizers by fragment‐based NMR screening. An initial screening identified cyclobutane scaffold **45** (Figure 22) with affinity towards the N‐terminal domain of apoE4. In addition, the scaffold stabilizes apoE4 and affects the kinetics of liposome breakdown. Next, the authors aimed to enhance its potency by structure based‐drug design. An X‐ray co‐crystal structure of **45** bound to apoE4 showed its binding mode and possible positions for potency enhancement. Besides other binding interactions, the cyclobutyl ring fits in a hydrophobic subpocket generated by Trp26, Leu30 and Ala153. On this basis the authors chose to extend off the central phenyl ring on the *meta* position. Addition of an unsubstituted phenyl ring already resulted in a 4‐fold potency increase, most likely due to increased van der Waals contact. After more SAR studies compound **46** (Figure 22) was identified, bearing hydrogen bonding capable groups, engaging in hydrogen bonds with Asp35. This lead showed similar favorable apoE4 protein stabilization but at a 5‐fold lower concentration.

### 2.4 Cyclobutanes in obesity research

**Figure 23:**
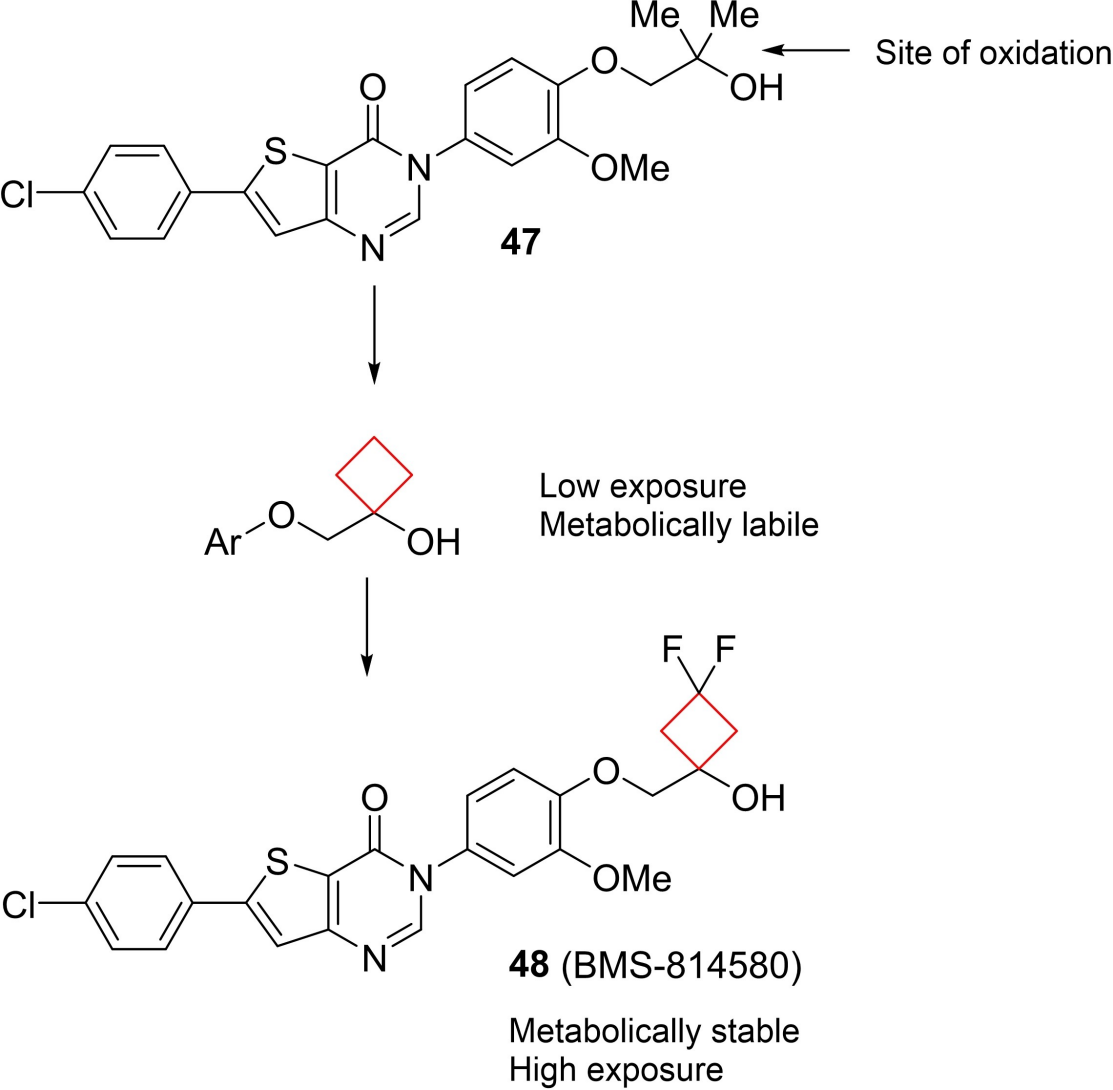
MCHR1 inhibitors **47** and its transition to more robust and potent lead compound **48**.

Compound **48** (BMS‐814580) is a potent and selective melanin concentrating hormone 1 (MCHR1) inhibitor, an enzyme largely involved in energy homeostasis. Inhibition of MCHR1 has been shown to be pharmacologically relevant to decrease obesity in animal studies.^[93]^ Ahmad and co‐workers^[34]^ aimed to eliminate toxic effects of metabolites of the previously reported^[94]^ lead **47** by improving its metabolic stability as it was found that the *gem*‐dimethyl substituent was prone to *in vivo* metabolic oxidation. Initially, a cyclobutyl substituent was installed with non‐satisfactory results, showing insufficient exposures. The difluorinated analogue **48** of the cyclobutyl drug was then synthesized to lower the cyclobutyl's susceptibility to metabolic oxidation and indeed showed major improvements in exposure and metabolic stability as depicted in Figure 23.

**Figure 24:**
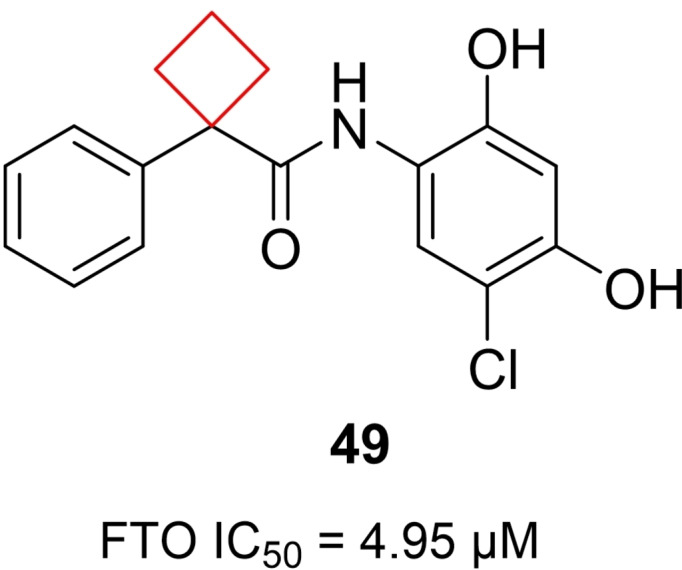
FTO inhibitor **49**.

He et al.^[95]^ set out to find small‐molecule inhibitors of the fat mass and obesity associated protein (FTO). FTO has been linked to obesity,^[96]^ but much of its biology remains unknown. A virtual screening was performed to identify small‐molecule inhibitors and led to multiple catechol derivatives connected through a cyclobutyl linker to another aromatic substituent. These compounds did not yield the desired profile. Therefore, the scaffold was changed from catechol to resorcinol, in which the geometry of the alcohols was reasoned to mimic the substrate 3‐methyl‐adenine better and thus improve binding. These studies resulted in the optimized compound **49** (Figure 24). Binding studies revealed that a combination of hydrogen bonds and van der Waals forces are responsible for its specific recognition. The cyclobutylphenyl moiety of **49** establishes extensive and close hydrophobic contacts with the non‐conserved antiparallel β‐sheet of FTO, yielding a potent and novel FTO inhibitor.

**Figure 25:**
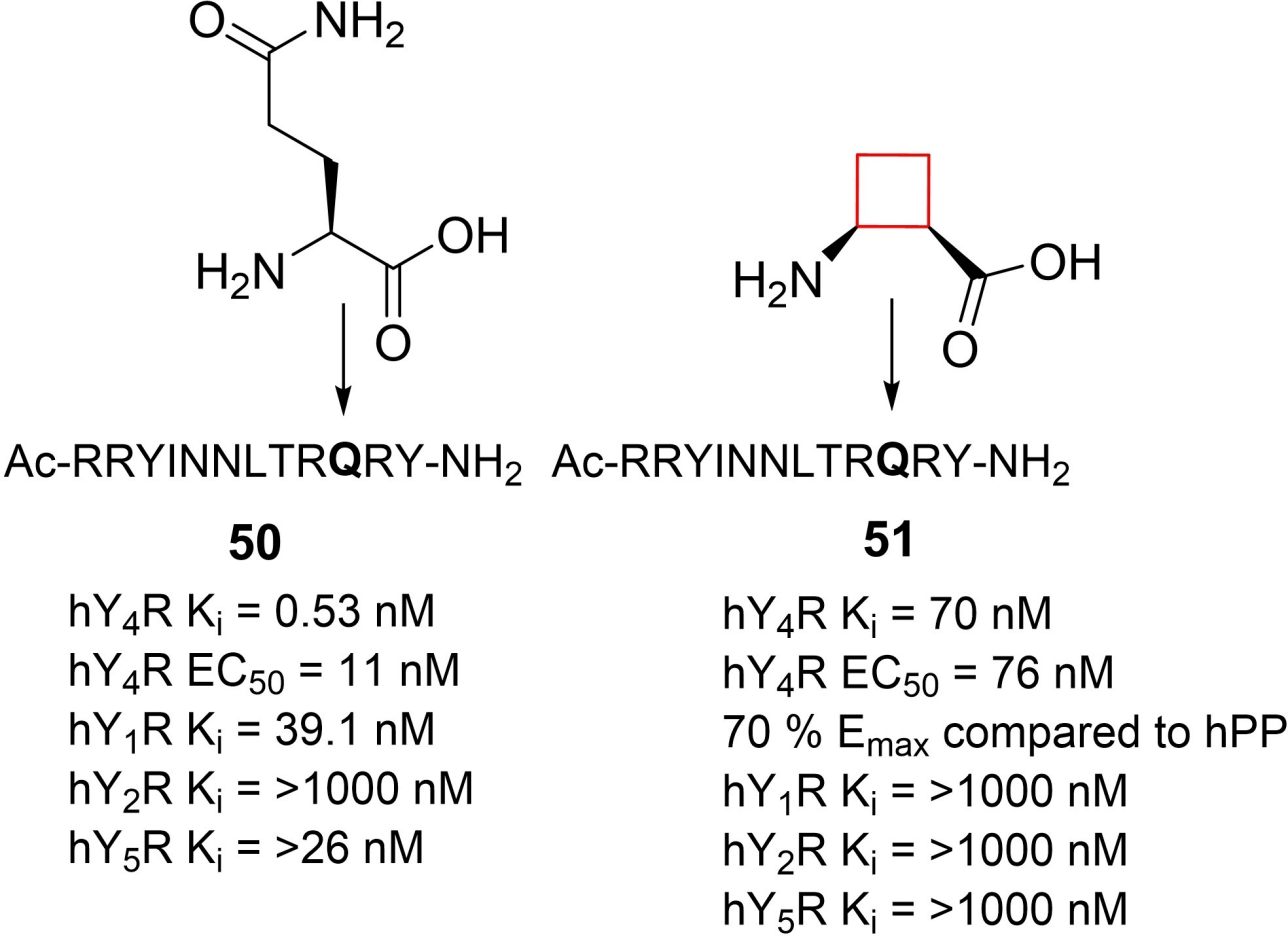
Y_4_R inhibitor **51** and natural peptide **50**.

G‐protein coupled receptor subtype Y_4_R selective agonists have been proposed as anti‐obesity agents.^[97]^ Natural neurohormone ligands of the human family of Y_x_R (x=1, 2, 4, 5) G‐protein coupled receptors are neuropeptide Y (NPY) and pancreatic polypeptide (PP). Potent and selective agonists and antagonists are available for all except for the Y_4_R subtype.^[98]^ Berlicki and co‐workers^[99]^ aimed to identify potent and selective small‐molecule Y_4_R inhibitors. The natural peptide hPP (**50**) is a Y_4_R ligand with unsurpassed affinity and agonistic potency (K_i_ 0.53 nM, EC_50_ 11 nM), albeit that it can also activate Y_1_R and Y_5_R. The authors set out to modify one of Y_4_R's natural ligand agonists to enhance its selectivity towards being a more potent and selective agonist compared to the 2‐digit micromolar potent selective Y_4_R agonist known to date of publication. The authors installed unnatural cyclic cycloalkane scaffolds into the peptide chains of **50** by replacing a glutamine residue, to induce enhanced selectivity and potency, but also to improve the stability towards protease degradation. A cyclopentyl candidate showed the most favorable profile, but cyclobutyl candidate **51** (Figure 25) also exhibited double digit nanomolar potency towards Y_4_R, as well as selectivity over the other subtypes of the Y_4_R family.

**Figure 26:**
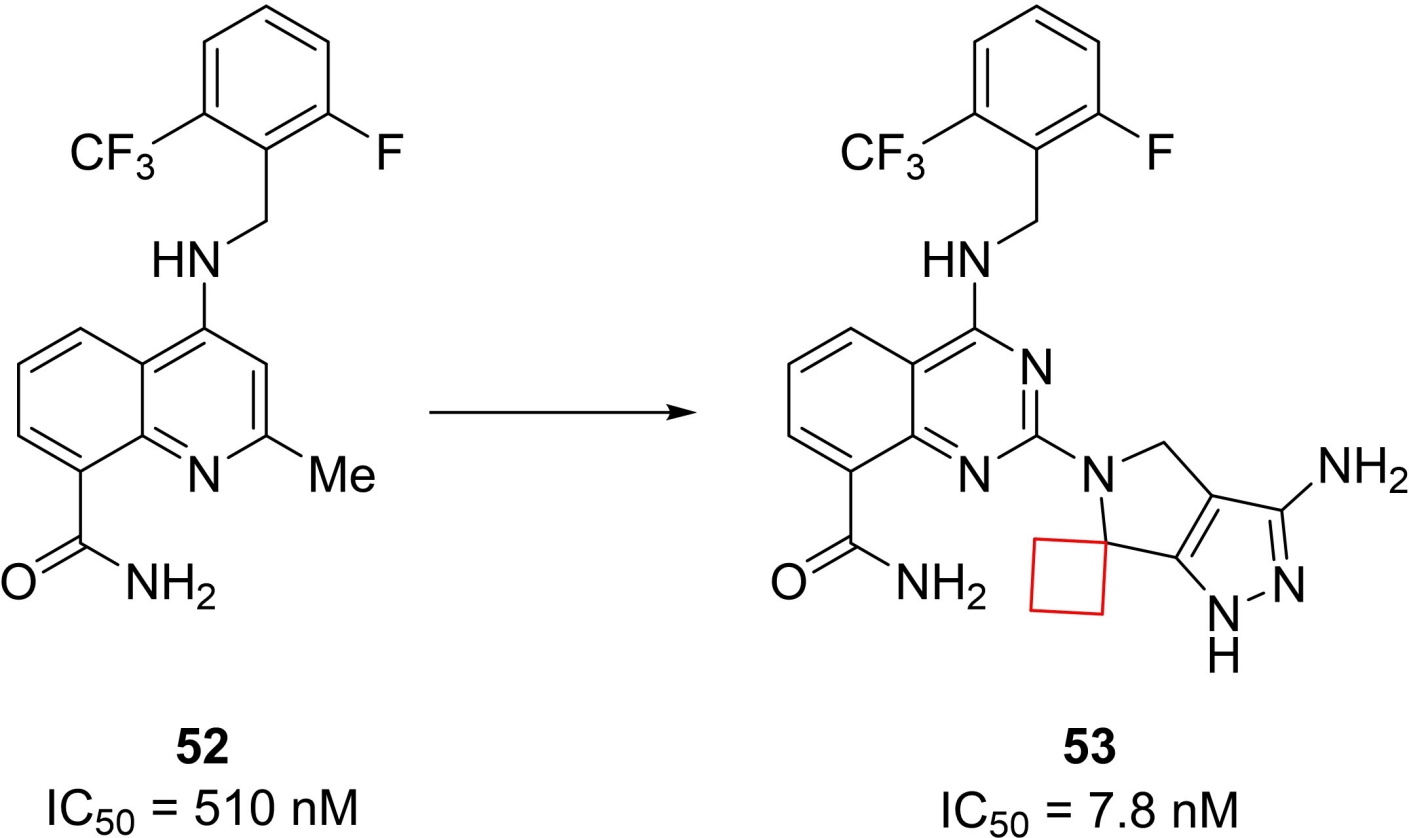
CD38 inhibitors **52** and **53**.

Clusters of differentiation 38 (CD38) is a type II membrane glycoprotein mostly located in immune cells but also expressed in bone and other major organ tissues such as liver, intestine, pancreas, muscle and brain.^[100]^ Besides having receptor functions, it shows enzymatic activity towards redox factor nicotinamide adenine dinucleotide (NAD). As it is consumed, it is converted by CD38 to cyclic adenosine diphosphate ribose (cADPR) and ADPR. CD38 therefore plays a central role in NAD cofactor modulation. Artificially keeping NAD levels high by CD38 inhibition could have a positive effect on metabolic diseases such as obesity. Previously identified quinoline‐8‐carboxamide CD38 inhibitor **52** raised NAD levels in a diet‐induced obese mouse model with acceptable pharmacokinetics.^[42]^ However, the authors intended to increase its potency further as well as reduce liabilities such as hERG inhibition. CD38 X‐ray co‐crystal structures of previously identified inhibitors revealed additional space in the binding pocket at the 2‐position of the quinoline that could be explored for the desired pharmaceutical properties. When exploring 1,4,5,6‐tetrahydropyrrolo[3,4‐*c*]pyrazole substituents at this position, the authors initially inserted geminal substituents to avoid inserting a chiral center. A *gem*‐dimethyl substituent at this position showed acceptable potencies. However, spirocyclic cyclopropyl and cyclobutyl derivatives appeared most potent for enzyme inhibition. Even though the cyclobutyl derivative **53** (Figure 26) did not exhibit the most ideal properties, the reduction of planarity by the cyclobutyl introduction might decrease the crystal lattice energy of the solids, resulting in an enhanced solubility according to the authors.

### 2.5 Cyclobutanes in antiviral compounds

**Figure 27:**
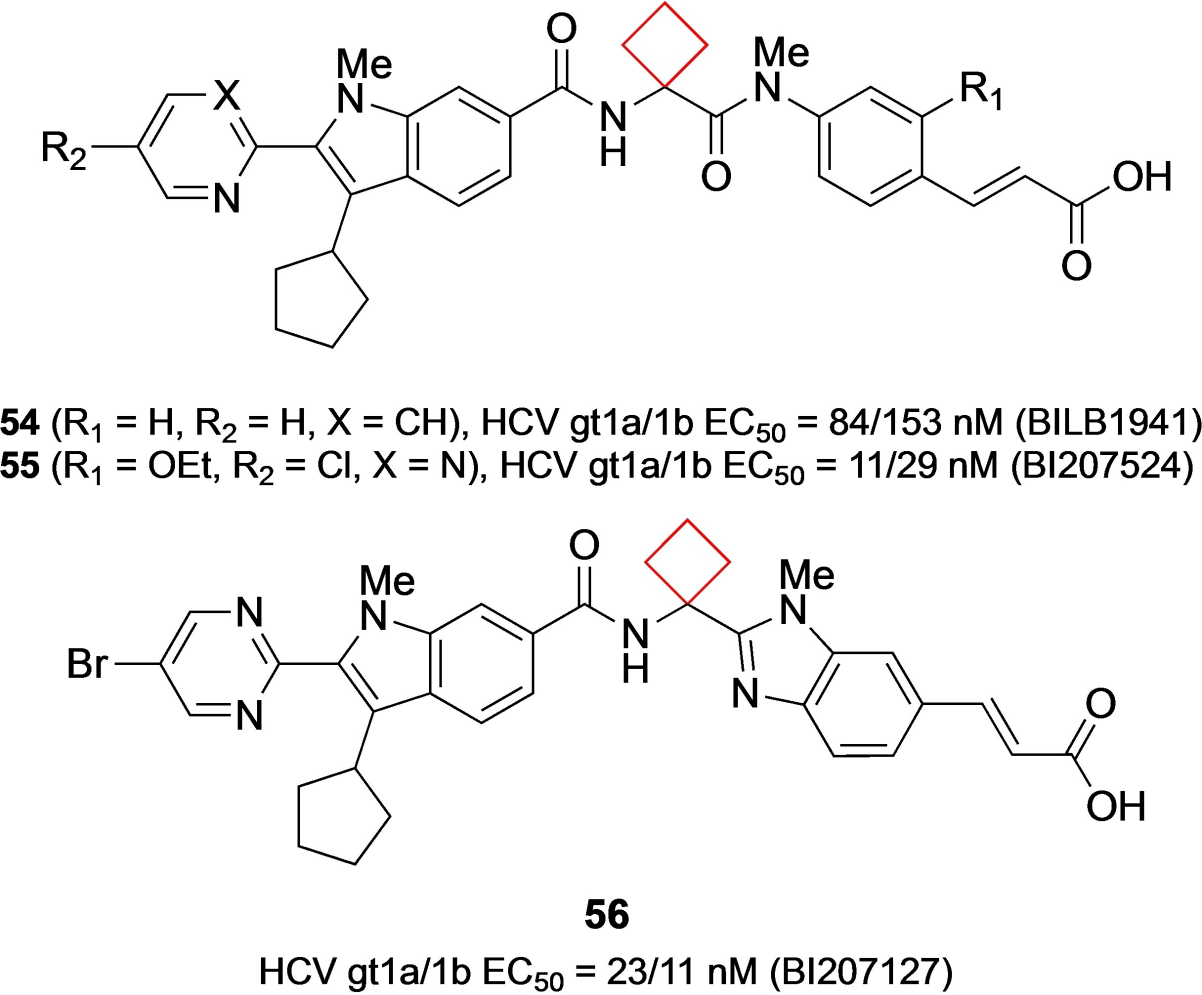
NS5B thumb pocket 1 inhibitors **54**, **55**, and **56**.

Hepatitis C virus (HCV) is a pathogen affecting millions of people worldwide.^[101]^ Numerous efforts to produce antivirals have been made, but room for improvement is still vast. Beaulieu and co‐workers^[102]^ have actively investigated allosteric inhibitors of the main viral RNA polymerase non‐structural protein 5B (NS5B) in one of its pockets “Thumb 1” vital to its RNA synthesis abilities. In an effort to late stage optimize known inhibitors the authors found that substituted indole–cinnamic acid derivatives were optimal for this goal (Figure 27). When optimizing the diamide linker between the two moieties it was found that cyclobutyl or cyclopentyl had significantly improved absorption irrespective of the substituents on the indole scaffold. While a *gem*‐dimethyl linker had improved cell‐based potency, the cyclobutyl linker showed improved oral bioavailability and an overall balanced profile of potency, *in vitro* ADME properties and *in vivo* rat exposure. This observation was found consistent across numerous indole–cinnamic acid derivatives and allowed for development of clinical lead compounds **54** (BILB1941) and **55** (BI207524) (Figure 27 *)*. Compound **54** was submitted to clinical trials, but no further studies were performed after phase II. Despite its attractive potency compared to **54**, compound **55** was not submitted to clinical trials since in preclinical studies was found that a genotoxic aniline metabolite formed in human and rat liver microsome assays. In later optimization efforts, LaPlante et al^[103]^ further elaborated on these chemotypes. Continued conformational restrictions were applied in the molecule guided by NMR, producing new scaffolds that showed optimized properties. The main change applied was cyclization of one of the diamides to a 1‐methylimidazole moiety as in compound **56** (BI207127). This compound was submitted to clinical trials and is currently under investigation in phase III.

**Figure 28:**
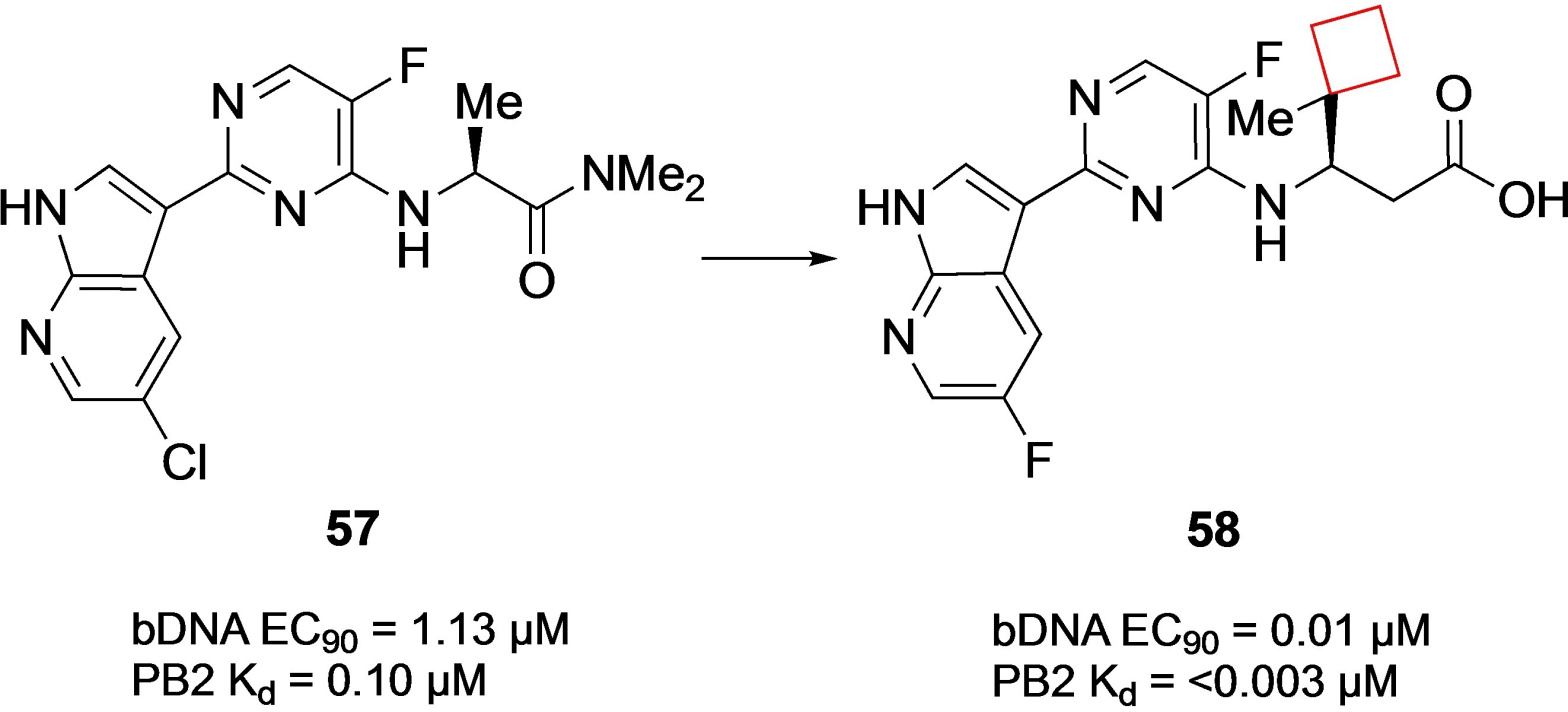
PB2 inhibitors **57** and **58**.

The influenza virus remains a global challenge as seasonal outbreaks keep afflicting large human populations. Additionally, pandemic outbreaks such as the 2009 H1N1 swine flu or the H5N1 bird flu are examples of the potential severity of the influenza strain and therefore drug discovery efforts remain of high interest. Targeting viral replication pathways remains a suitable strategy for small‐molecule inhibitors. The current standard of care for influenza infections utilizes this in the form of neuraminidase inhibitors.^[104]^ Farmer et al.^[28]^ previously identified^[105]^ the novel 7‐azaindole **57** (Figure 28) inhibitor from a phenotypic cell protection assay screen showing survival benefits in a mouse model when administered 48 h post infection.^[105]^ These inhibitors inhibit the influenza polymerase‐B2 (PB2) heterotrimeric complex essential to viral RNA replication. In addition to replacing the chloride for a fluoride atom in previous research, optimization efforts focused on the alanine diethylamide side chain. After docking and X‐ray studies on the PB2 binding pocket the authors conjectured that synthesizing derivatives containing *tert*‐butyl side groups would fill the hydrophobic pocket defined by three phenylaniline residues. The best analogue showed two‐digit nanomolar affinities, but the authors suggested based on the X‐ray structures of the *tert*‐butyl analogues that the pocket could be filled by even larger substituents. After numerous iterations, it was observed that cyclohexyl derivatives showed promising activities, leading to the synthesis of more cycloalkyl substituents. The methyl‐spirocyclobutyl compound appeared the most potent one, while removal of the methyl led to a 100‐fold decrease in activity, displaying the stringent balance in shape and size for filling this hydrophobic pocket. This result led to a potent cyclobutyl containing candidate **58** (Figure 28) with a potential as a therapeutic.

### 2.6 Cyclobutanes in antidiabetes compounds

**Figure 29:**
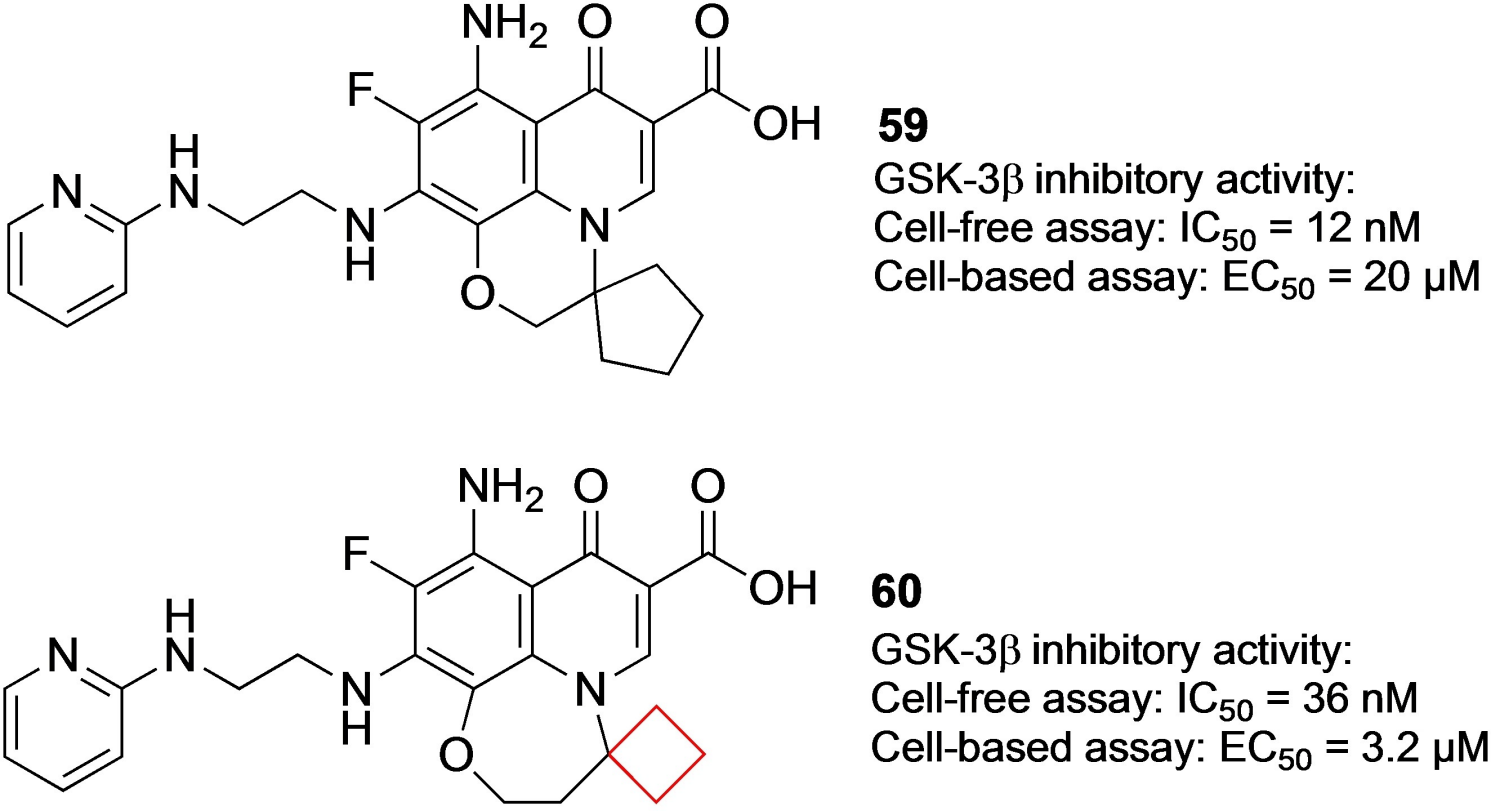
GSK‐3β inhibitors **59** and **60**.

Glycogen synthase kinase 3 (GSK‐3) is a serine/threonine protein kinase that phosphorylates and inactivates glycogen synthase, limiting the formation of glycogen. GSK‐3 activity and expression are found to be higher in type 2 diabetics.^[106]^ Therefore the inhibition of GSK‐3 in type 2 diabetes patients might be a reasonable target to find pharmaceuticals effective against this condition.^[107]^ Seto and co‐workers^[108]^ previously identified the potent and selective GSK‐3β quinolone inhibitor **59** (Figure 29). These inhibitors, however, were not as potent in cell‐based tests. The authors hypothesized that this lack in potency was because of insufficient cell permeability. They then merged the quinolone moiety of the previous inhibitor **59** with another inhibitor known in literature to arrive at a 6–6–7 quinolone ring system. This compound was subjected to a SAR study also involving a spirocyclic substituent. It was concluded that cyclobutyl derivative **60** (Figure 29) exhibited GSK‐3β inhibitory activity in both cell‐free and cell‐based assays. In addition, compound **60** decreased the plasma glucose concentration in a dose‐dependent manner in an oral glucose tolerance test in mice. Based on these promising results, this compound will be subjected to further biological testing.

**Figure 30:**
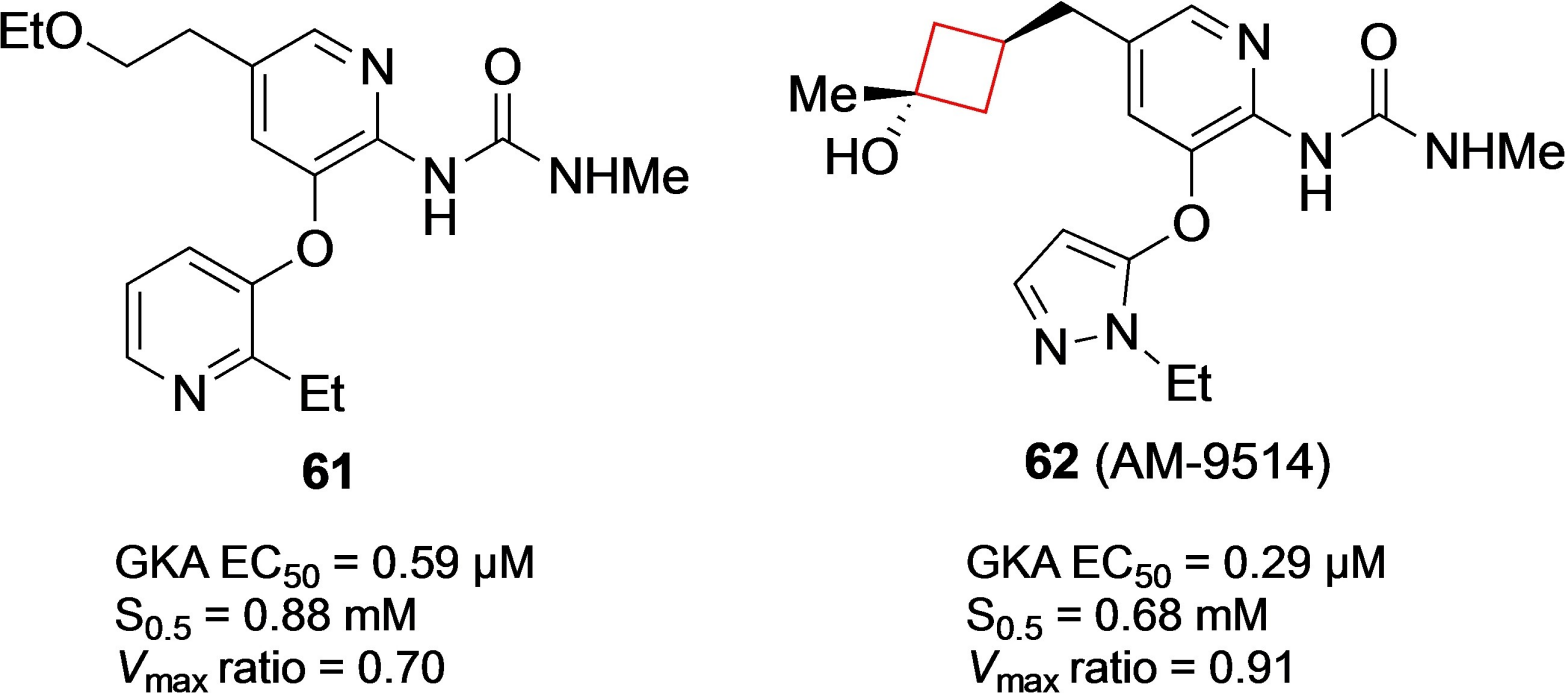
GKAs inhibitors **61** and **62**.

Intracellular enzyme glucokinase (GK) is present in both liver hepatic and β‐cells and is responsible for the first step in glucose utilization being the conversion of glucose into glucose‐6‐phosphate.^[109]^ In both tissues GK plays a role in regulating glucose utilization and production.^[110]^ Glucokinase activators (GKAs) influence glucose binding (*K*_m_ or S_0.5_) and the kinetic profile (*V*_max_) of the phosphorylation reaction.^[111]^ Therefore GKAs play a central role in regulating blood glucose concentrations and therefore can be employed as possible therapeutics for type 2 diabetes.^[112]^ Du et al.^[40]^ previously identified^[113]^ a series of GKAs inhibitors and pursued the identification of a structurally distinct series. While doing so, the authors aimed to keep S_0.5_>0.6 mM and 0.8<*V*_max_<1.3 based on data analysis to mitigate adverse effects. The authors pursued a methyl urea‐substituted pyridine series, including compound **61** (Figure 30). After some initial SAR studies, the authors also included cyclic alcohols at C‐5 as tertiary alcohols had been identified to prevent secondary metabolism. In addition, the introduction of cyclic moieties may decrease the degrees of freedom in the molecule, resulting in improved pharmacokinetic properties.^[114]^ Cyclohexanols showed some potency but outside the *V*_max_ range set by the authors. The values were more encouraging for cyclobutanols. A methyl was added to create a tertiary alcohol, of which the *trans*‐variant **62** (AM‐9514) (Figure 30) was most potent and in the desirable parameter range. It showed favorable *in vitro* properties as well as desirable pharmacokinetics in mouse and dog.

### 2.7 Cyclobutanes in miscellaneous disease areas

**Figure 31:**
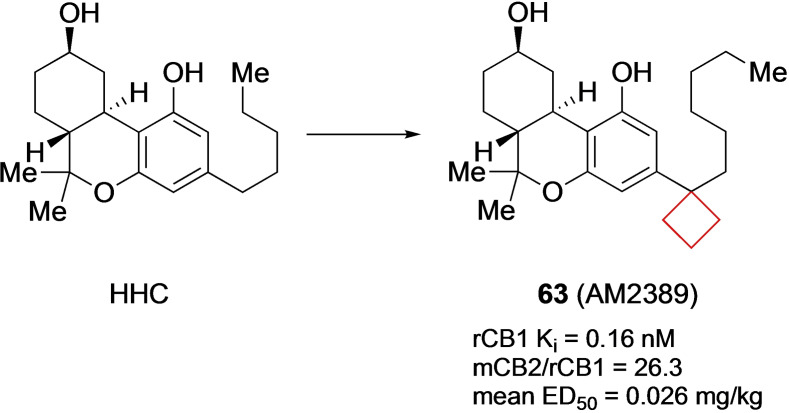
CB1 and CB2 inhibitors HHC and **63**.

**Figure 32:**
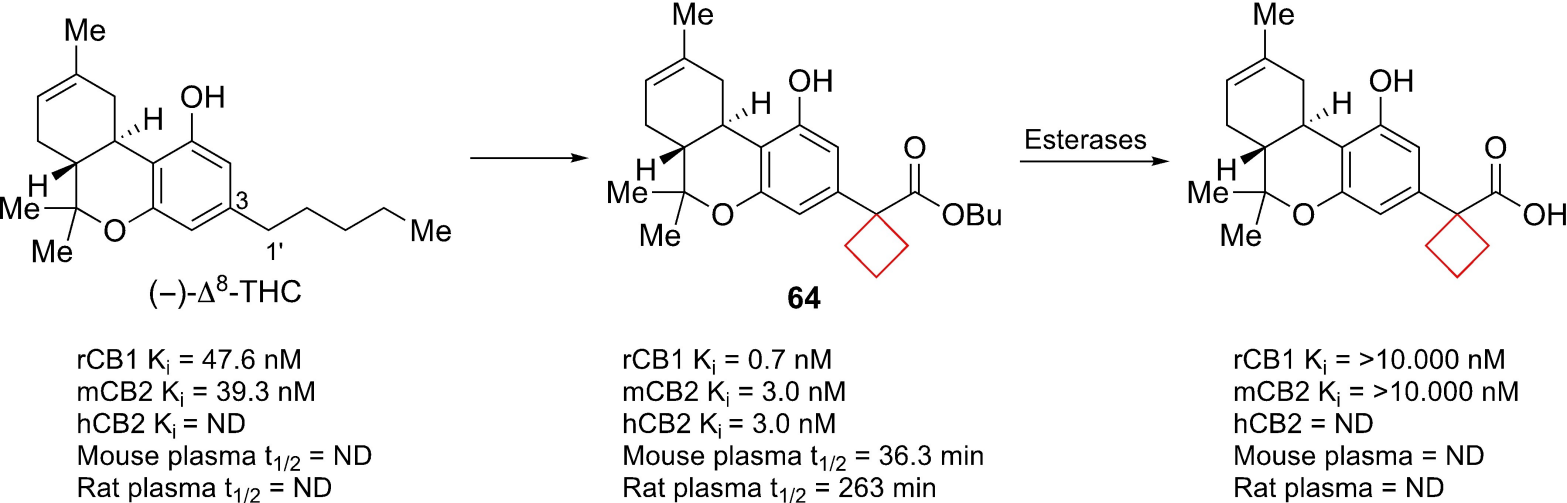
CB1 and CB2 agonist **64**, its de‐esterified analogue and (−)‐Δ^8^‐THC.

Compound **63** (AM2389) is a selective cannabinoid 1 (CB1) agonist. Modulation of the CB1 and CB2 receptors is a possible treatment for conditions such as pain and inflammation.^[115]^ It was previously identified that the phenolic hydroxyl and the lipophilic side chain are pharmacophores within the (−)‐Δ^8^‐tetrahydrocannabinol (THC) (Figure 32) core structure that determines its potency. Nikas and co‐workers^[29]^ investigated the effect of modifying the C3 side lipophilic tail on hexahydrocannabinol (HHC) derivatives towards their potency and selectivity. The authors previously identified C1′ cyclic substituents to be useful moieties for achieving potency in (−)‐Δ^8^‐THC derivatives. Using the same strategy, numerous derivatives bearing cyclopentyl or cyclobutyl groups on the C1′ position were synthesized to assess the effect of conformational restriction towards potency and selectivities of HHC derivatives. According to their hypothesis, the cyclopentyl and cyclobutyl rings increased the potency towards CB1 and CB2 receptors significantly. The cyclopentyl moiety was most potent, though the cyclobutyl still had a high potency, as well as high selectivity (Figure 31). The authors conducted a modelling study of **63** in implicit water for CB1 binding. It was found that the cyclobutyl ring could engage in optimal interactions at the putative receptor‐binding site. *In vivo* experiments showed that **63** has a slow onset (60–90 min at 0.1 mg/kg in rats), a long duration of action, and a high potency in assays of antinociception and hypothermia. When compared to morphine, the analgesic action of **63** was 100‐fold higher.

Compound **64** is an agonist of CB1 and CB2 receptors. Seeking to expand the medicinal toolbox of cannabinergic drugs, Sharma et al.^[36]^ developed novel (−)‐Δ^8^‐THC derivatives. The authors aimed to solve the fact that cannabinoid receptor pharmaceuticals metabolites often show adverse or unpredictable side effects.^[116]^ It was speculated that if a biologically labile substituent was introduced on the 3‐side tail of (−)‐Δ^8^‐THC it would predictably and controlled metabolized in a way that can be chemically tuned. Using this so‐called soft‐drug approach, an ester was introduced at the 2′‐position of the alkyl tail. Using modifications at the 1′‐position the lability could be tuned. This position was functionalized with methyl (both *R* and *S* diastereoisomers), *gem*‐dimethyl and a cyclobutane substituent. Though the potencies towards CB1 and CB2 were comparable to other potent CB1 and CB2 agonists, the cyclobutyl scaffold showed the most favorable kinetics. Its mouse and rat plasma half‐lives were significantly longer compared to the other derivatives, ensuring a larger therapeutic window. Molecular modelling studies revealed that the cyclobutyl ring optimally occupied a putative subpocket of the CB1 receptor. The larger van der Waals radius of the cyclobutyl group is proposed to also hinder its enzymatic cleavage by esterases (Figure 32). In addition, the esterase cleavage product of **64** has no significant affinity for CB receptors. In this way the cyclobutyl ring was used both as a tool to fill the enzymatic pocket as well as slow down the enzymatic metabolism.

**Figure 33:**
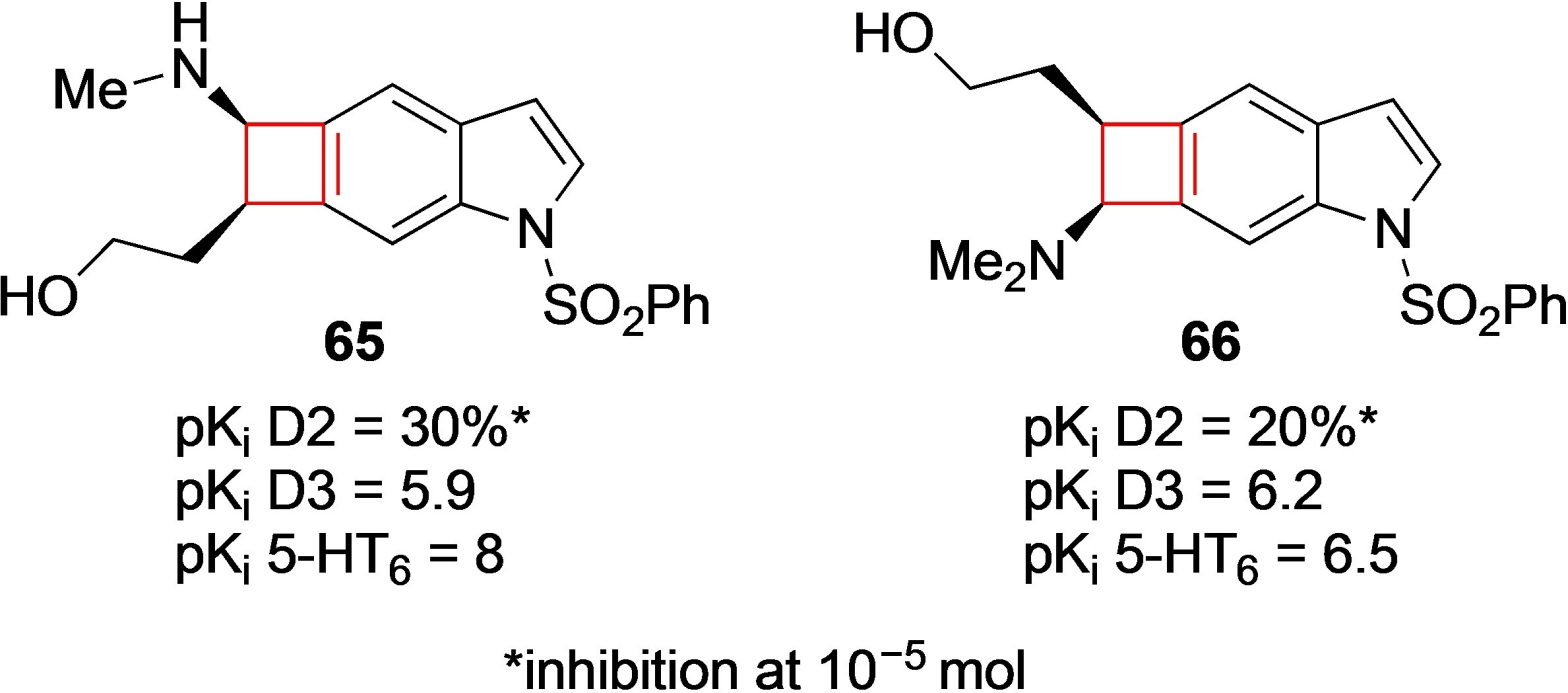
5‐HT_6_ receptor ligands **65** and **66**.

Saavedra and co‐workers^[117]^ sought to identify novel antipsychotics to battle schizophrenia. Current antipsychotics are limited in their ability to combat some of the negative and neurocognitive effects of schizophrenia.^[118]^ The authors aimed to design potent blockers that showed selectivity on dopamine receptor 3 (D3R) over D2R receptors as well as having 5‐HT6R blocking potency. Based on previous findings in literature, the authors designed cyclobutaindole derivatives hypothesizing that these compounds would exhibit the desired pharmacological profile. Having executed numerous SAR studies, compounds **65** and **66** (Figure 33) showed optimal results. These lead compounds behaved as 5‐HT6R ligands as well as exhibiting D3R selectivity over D2R.

**Figure 34:**
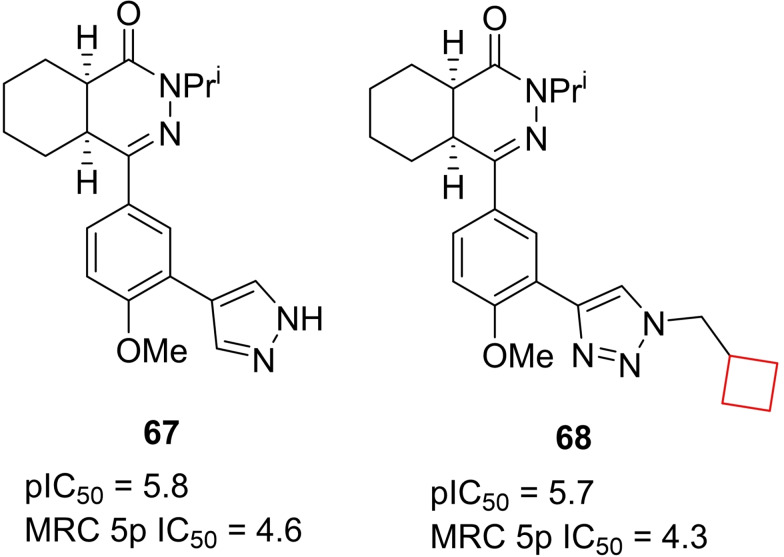
Anti‐leishmanial inhibitors **67** and **68**.

Visceral Leishmaniasis (VL) is caused by *L. donovani* and *L. infantum*. The current treatment is effective, but far from ideal, since it expensive, accompanied by pain and often toxic side effects.^[119]^ Sijm et al.^[120]^ aimed to develop novel potent compounds showing anti‐leishmanial activity, but with a smaller risk of toxic side effects. In an initial library screening, hexahydrophthalazinone **67** was identified as a hit that formed the starting point for a SAR study. Although other candidates showed higher potency, candidate **68** (Figure 34) exhibited lower cytotoxicity towards human MR5‐C cells. This compound is a viable candidate for further studies and optimization on anti‐leishmanials.

**Figure 35:**
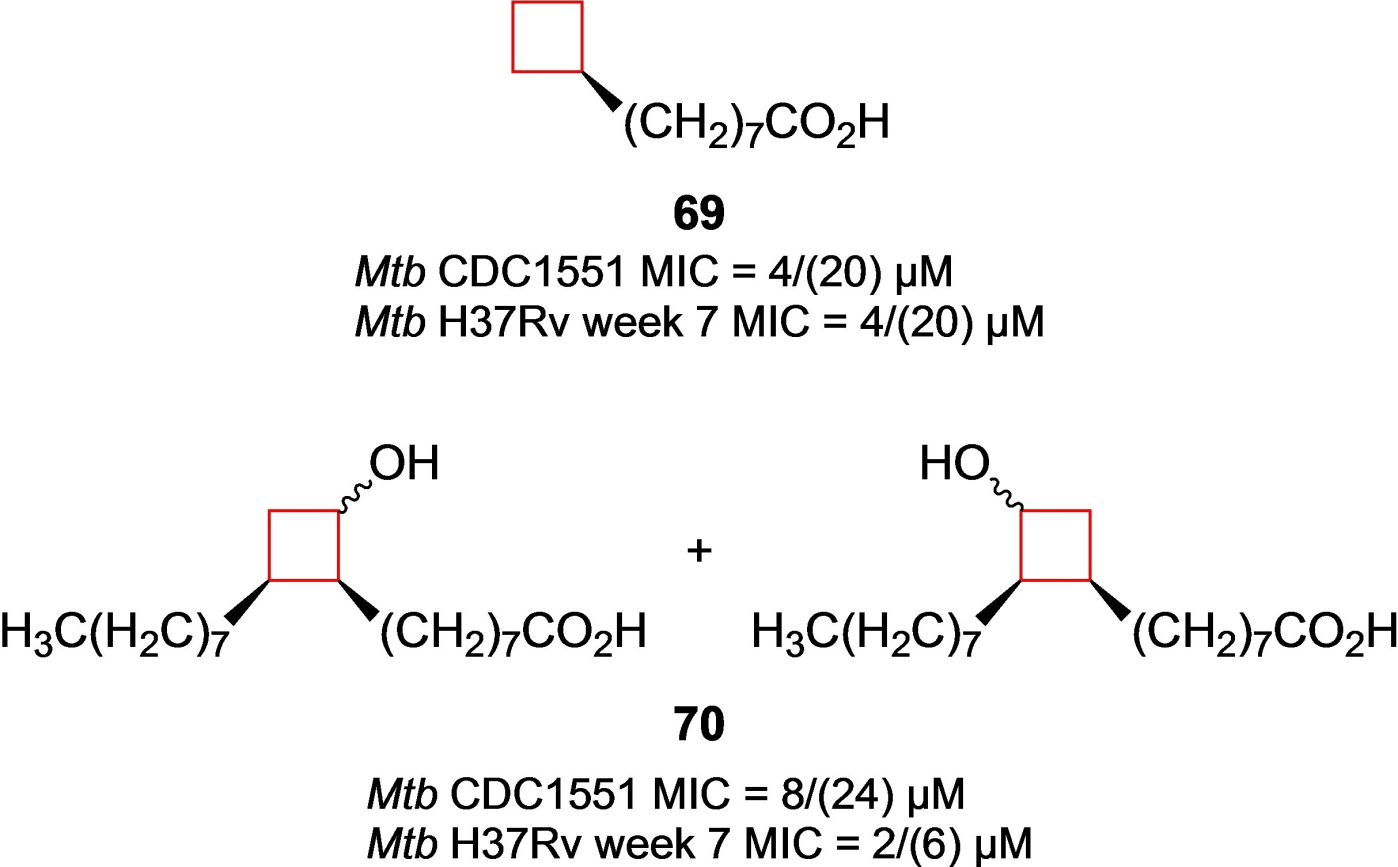
Mtb inhibitors **69** and **70**.

*Mycobacterium tuberculosis* (*Mtb*) is a bacterium responsible for tuberculosis (TB) and has a significant presence among human populations. Therapeutic agents for its treatment have been developed, but significant side effects are often observed during the long period that the patients have to take the medication.^[121]^ This results in unwanted consequences such as non‐compliance, relapses and the emergence of multidrug‐resistant strains.^[122]^ Therefore, novel therapeutics are constantly being developed and studied. The sturdiness and drug‐resistant properties of the mycobacteria is mostly because of the thick lipid cell wall that consists for a large part of mycolic acids. Existing *Mtb* treatments focus on inhibiting the enzymes responsible for the biosynthesis of mycolic acid. It is known that mycobacteria incorporate C_16_ and C_18_ fatty acids into their mycolic acid synthesis, as well as modified ones.^[123]^ Therefore, Sittiwong and co‐workers^[124]^ hypothesized that specifically functionalized fatty acids might hijack this pathway and limit mycobacterial growth. The authors synthesized carbocyclic derivatives of decanoic and oleic acids with different functionalities. The cyclobutane derivatives showed significant inhibitory anti‐mycobacterial activity against *Mtb*, **69** and **70** (Figure 35) appeared more active than clinically used TB drug *D*‐cycloserine (CDC1551 & H37Rv week MIC: 4/(39) and 8/(78) μM, respectively) and **69** proved equally, if not more potent than isoniazid (CDC1551 & H37Rv week MIC: 4/(29) and 8/(58) μM, respectively). In a similar study performed by the same group, Zinniel et al.^[125]^ identified that the same group of cyclobutane derivatives of decanoic and oleic acids exhibit similar behaviour towards *mycobacterium avium paratuberculosis (Map)*. While both C_10_ and C_18_ derivatives showed efficacy in the *Mtb* study, only C_18_ analogues showed significant activity efficacy against *Map*. Compounds **69** and **70** will therefore be a good starting point for biological evaluations and might be useful starting tools for development of therapeutics targeting *Mycobacterium* species.^[126]^

**Figure 36:**
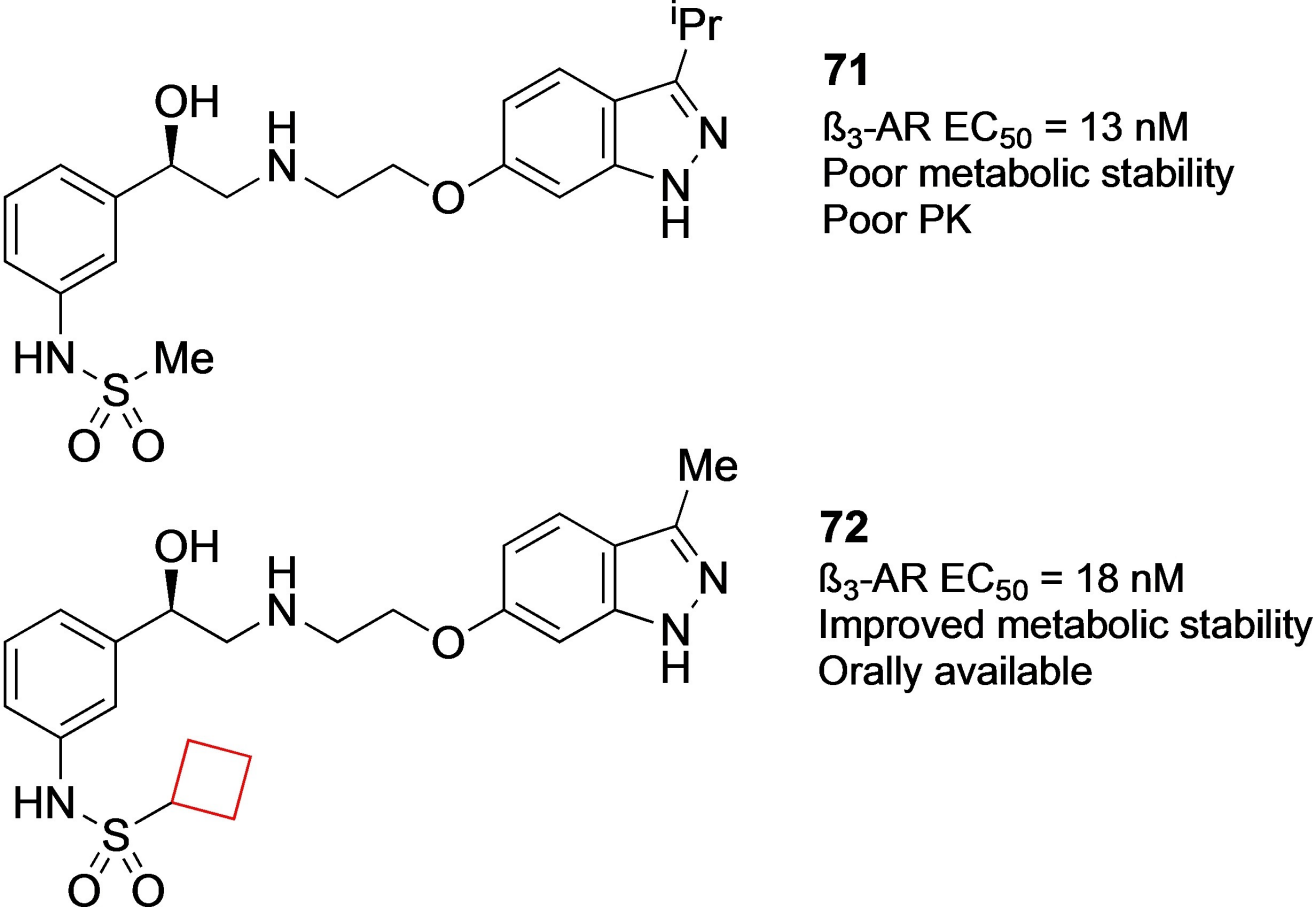
β_3_‐AR inhibitors **71** and **72**.

Compound **72** is a sulfonamide indazole derivative β_3_‐adrenergic receptor (AR) inhibitor. Wada et al.^[30]^ began searching for inhibitors of the β‐AR subfamily of enzymes as potential therapeutics against overactive bladder.^[127]^ Aiming to improve the PK properties of the previously reported lead **71** ^[128]^ as well as maintaining its selectivity, it was found that large alkane substituents on the sulfonamide sulfur resulted in higher potencies and selectivities. The cyclobutyl substituent gave the best balance between potency, selectivity and ADME properties. A molecular dynamics simulation was run to predict its binding mode. It was found that the cyclobutyl ring of **72** (Figure 36) fits the hydrophobic pocket of the target enzyme particularly well. This pocket was also partially responsible for the selectivity over α‐AR enzymes lacking a significant hydrophobic pocket compared to β_3_‐AR.

**Figure 37:**
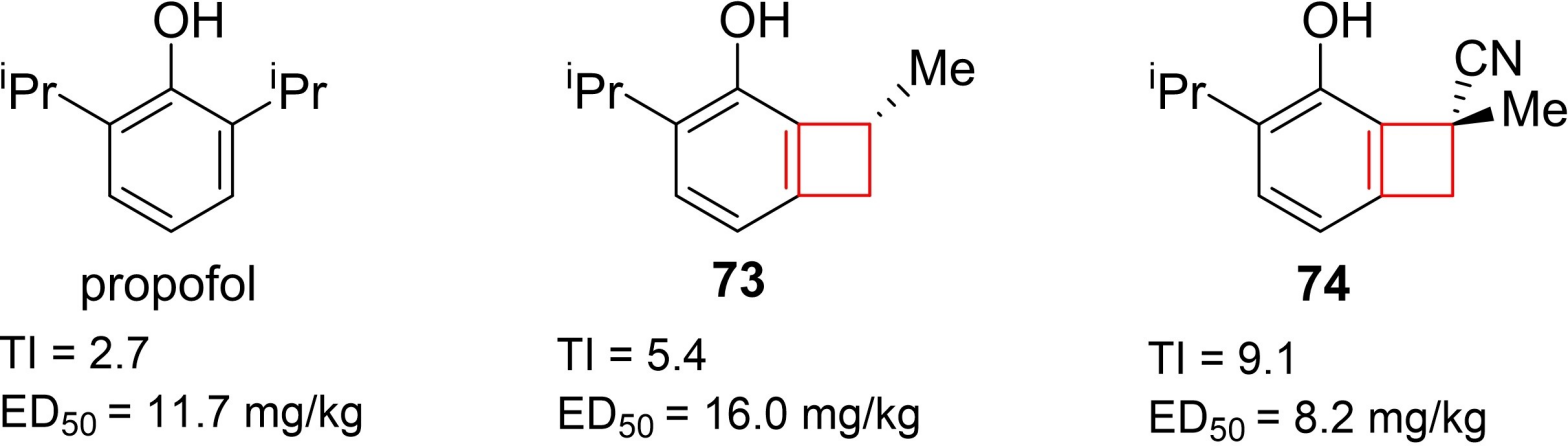
Propofol, cyclized analogue **73**, and optimized lead **74**.

Zhang and co‐workers^[41]^ attempted to reduce the negative side effects of propofol, the most widely used intravenous general anaesthetic in clinical use.^[129]^ Numerous propofol derivatives have already been synthesized to improve the overall profile. The authors noted that meta‐modification yielded suitable positions for modifications from literature.^[130]^ Installation of a methyl group on the meta‐position yielded a good therapeutic index as well as acceptable potency. Moving forward the authors decided to fuse the ortho‐ and meta‐substituents into a cyclobutane ring. This fusion, giving rise to compound **73**, both reduced the molecular weight and increased the scaffolds’ rigidity. Doing so already improved the therapeutic index 2‐fold with a retention in ED_50_ compared to propofol for the γ‐aminobutyric acid A receptor (Figure 37). From further SAR studies it was concluded that this moiety generally yielded drug candidates with improved therapeutic indices and comparable or enhanced ED_50_ values. Finally, introduction of a hydrogen bond acceptor on the tertiary carbon improved the pharmacological profile as well, yielding optimized structure **74** (Figure 37).

## 3 Conclusions

This review highlights the role of cyclobutyl rings in (pre)clinical drug candidates and is summarized in Table 1 below. Based on this review, researchers could use rational design for applications of a cyclobutane ring in small‐molecule drug development such as conformational restriction, increase in rigidity, filling of a hydrophobic pocket and directing key pharmacophores. Other uses of a cyclobutane ring such as improvement of metabolic stability, as an aryl isostere, improved solubility or affinity alteration may be less straightforward. The examples in this review do however illustrate its use in these areas. This potentially gives the medicinal chemist one more tool for tackling these challenges in the development of small‐molecule drug candidates in the future and in addition also potentially allows for greater insight into using a cyclobutane ring for these applications.

**Table 1:** Summary of contributions of a cyclobutyl ring towards drug properties discussed in this article.

Property	Contribution	Compound example, drug target and pharmacological area		
		Compound example	Drug target^[Ref.]^	Pharmacological area
Binding pocket exploitation	Fits well in hydrophobic pocket and directs nitrile group	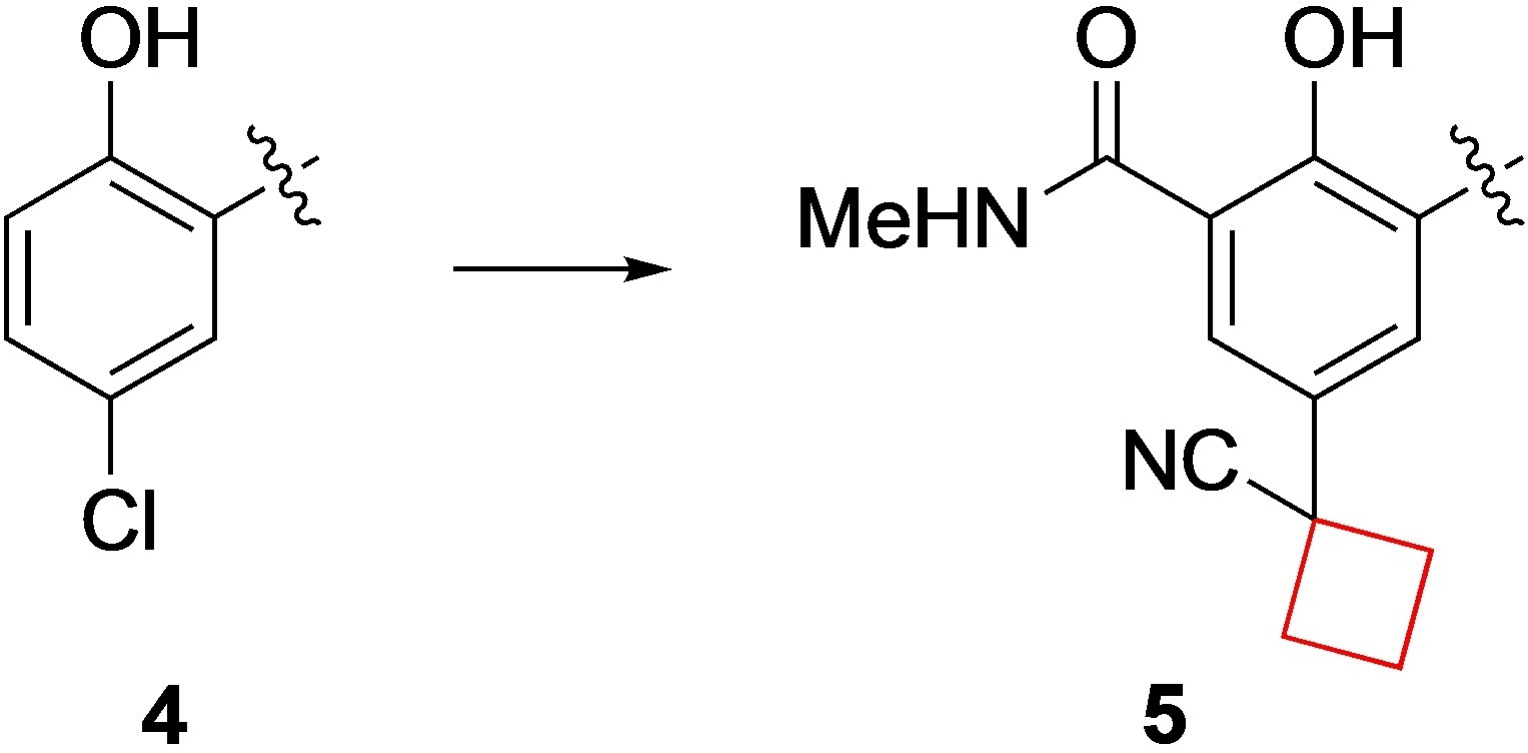	WDR5 inhibitor^[17]^	Cancer
	Fits well in hydrophobic pocket	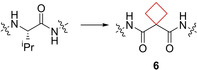	Cathepsin B substrate^[23]^	Cancer
	Directs triazole motif towards more efficient interactions of the pyrimidine scaffold	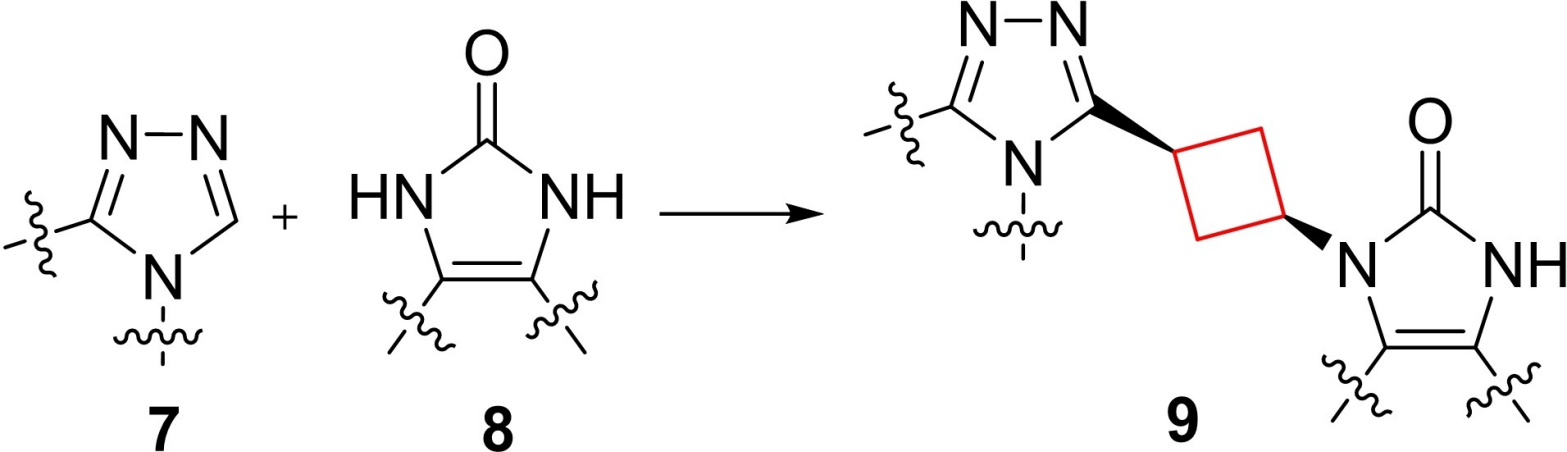	TNKS1/2 inhibitor^[18]^	Cancer
	Directs amine towards favourable interactions in binding pocket	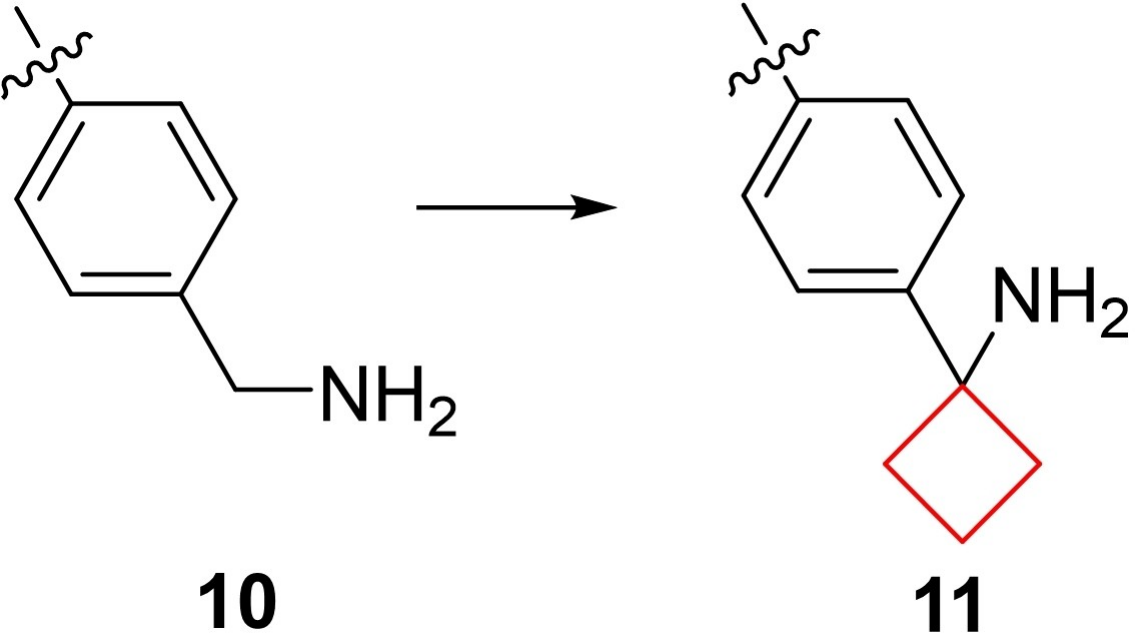	Allosteric AKT1/2/3 inhibitor^[19]^	Cancer
	Fits well in hydrophobic pocket driven by desolvation.	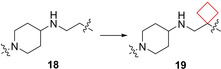	P97 ATPase inhibitor^[24]^	Cancer
	Puckered conformation allows for favourable interactions of sulphonamide in binding pocket	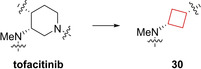	JAK1 inhibitor^[20]^	Autoimmune disease
	Cyclobutyl motif fits well in hydrophobic pocket	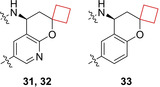	BACE1 inhibitor[^25^, ^26^]	AD
	Cyclobutyl motif directs amide towards favourable interactions	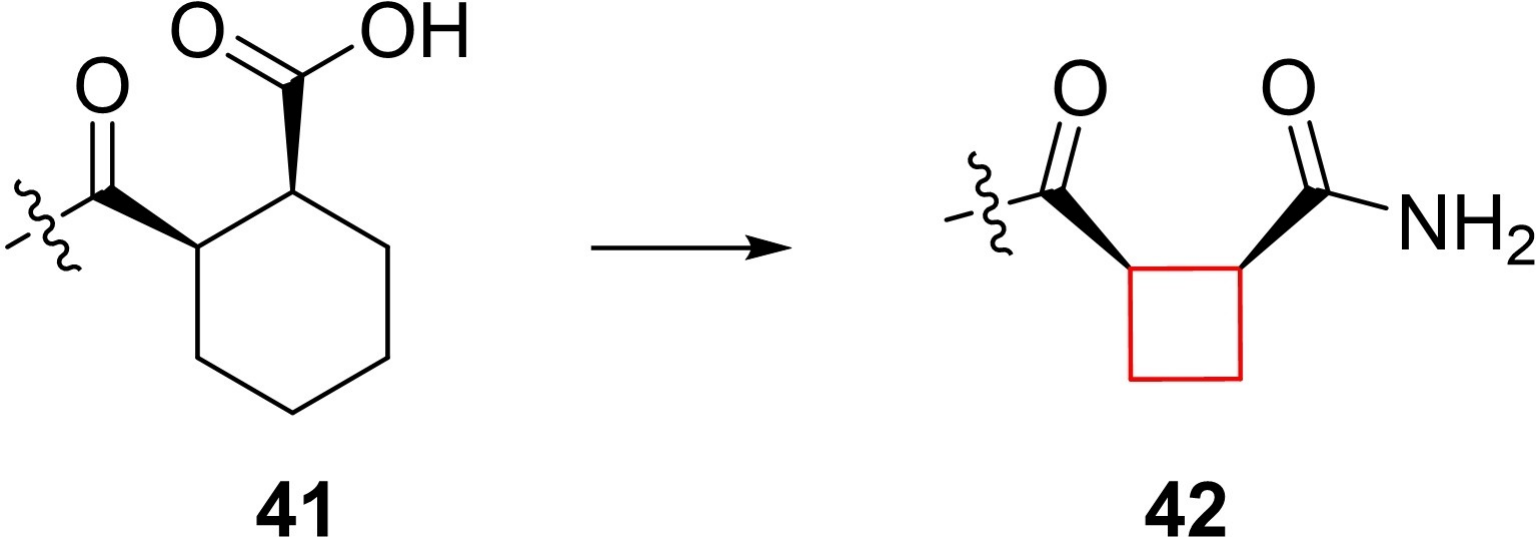	KEAP1/NRF2 interaction inhibitor^[21^	Parkinson's, Huntington's
	Cyclobutyl fits well in narrow binding hinge and directs substituents	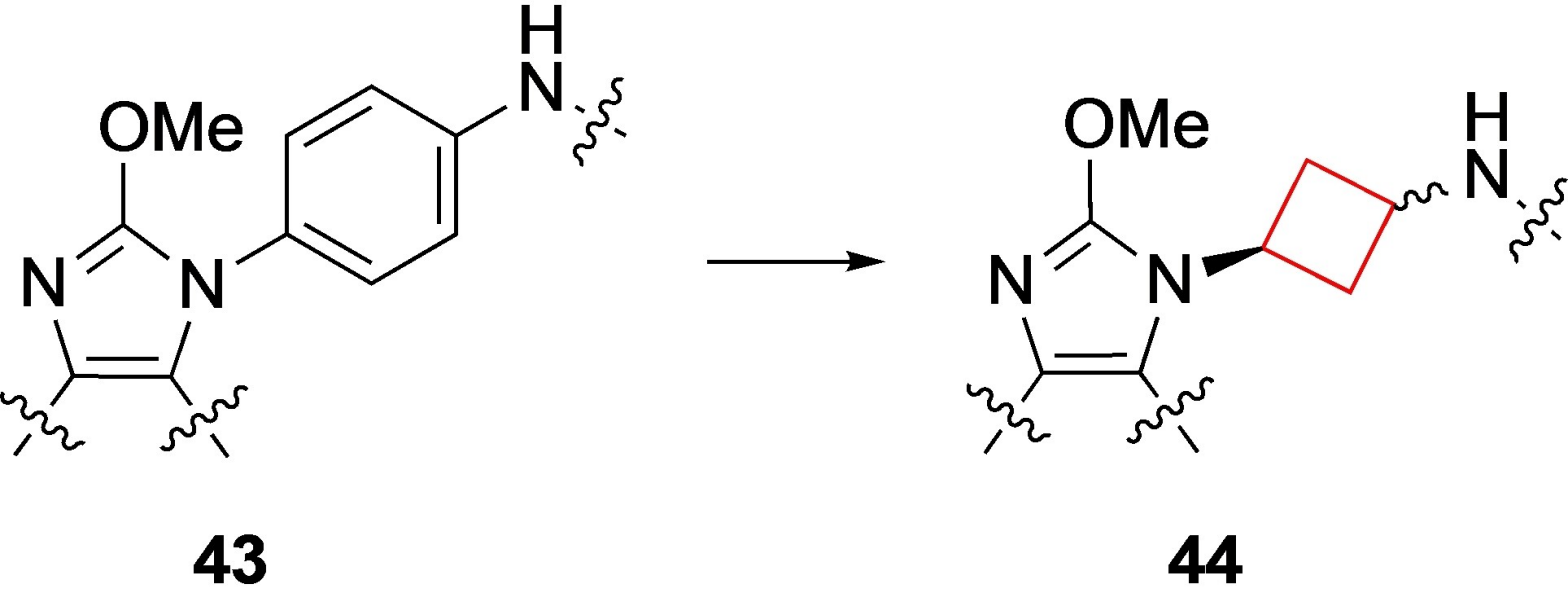	PDE10A inhibitor^[22]^	Schizophrenia
	Cyclobutyl fits well in hydrophobic binding pocket	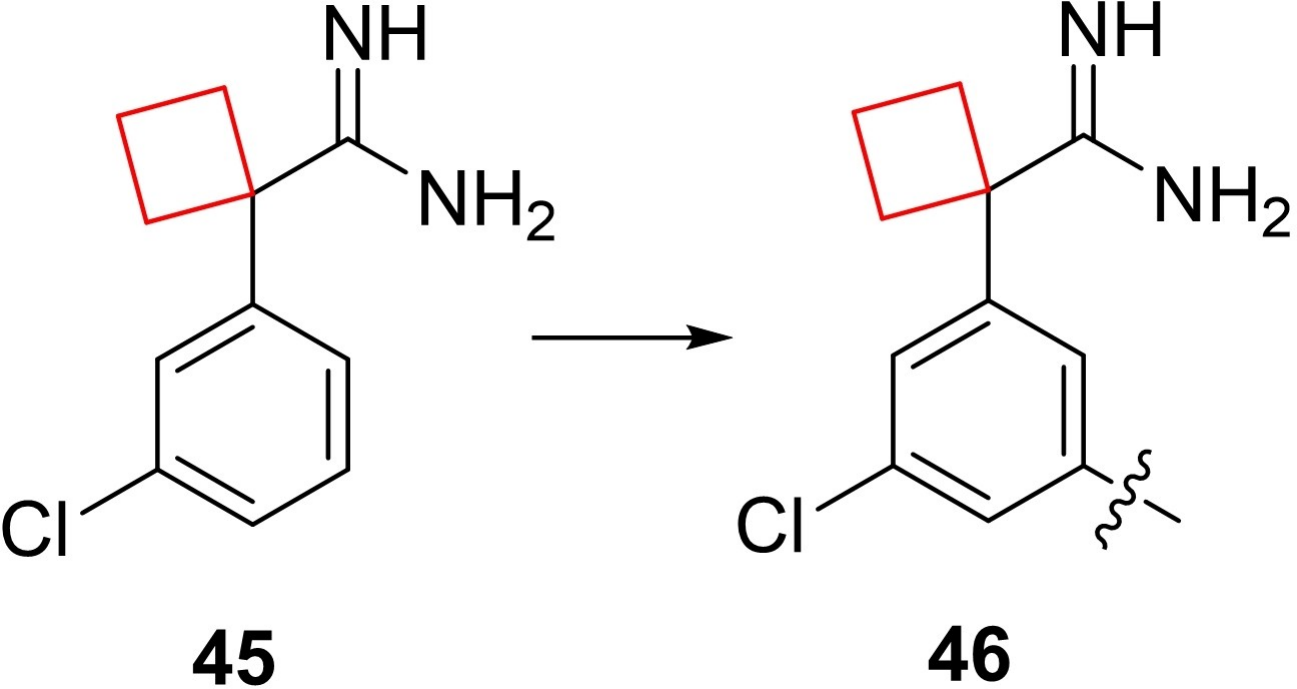	APOE4 stabilizer^[27]^	LOAD
	Cyclobutane ring establishes extensive van der Waals contact in binding pocket	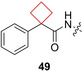	FTO inhibitor^[95]^	Obesity
	Methylcyclobutyl substituent fills hydrophobic pocket well	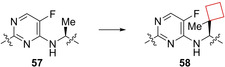	Influenza PB2^[28]^	Influenza virus
	Cyclobutyl engages in interactions in putative binding site	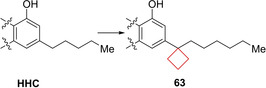	CB1 agonist^[29]^	Inflammation
	Fit hydrophobic pocket	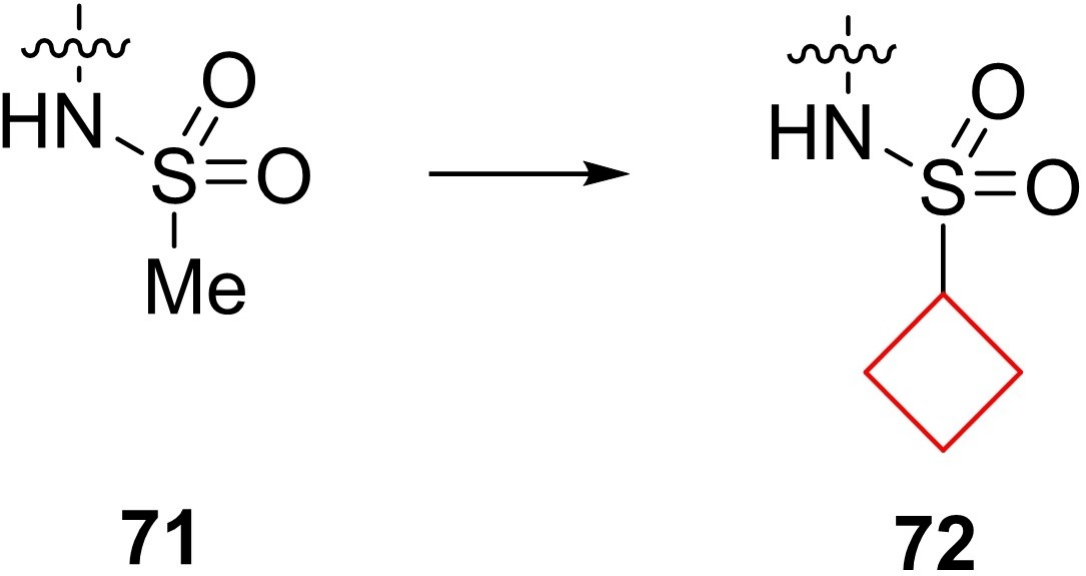	ß_3_‐AR agonist^[30]^	Overactive bladder
Chemical stability	Locks scaffold in biologically active cis‐conformation Inducing increased potency	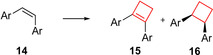	Tubulin polymerization inhibitor^[32]^	Cancer
	Locked into cis‐conformation but lower potency	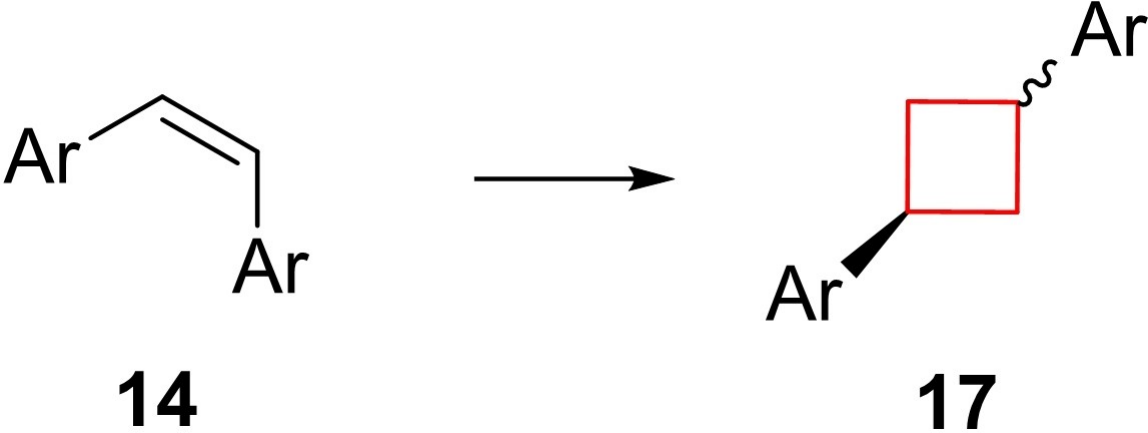	Tubulin polymerization inhibitor^[33]^	Cancer
	Prevents cis/trans isomerization of the alcohol	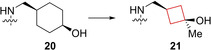	TTK inhibitor^[31]^	Cancer
Metabolic stability	Increased	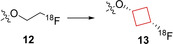	Cancer cells^[35]^	Cancer
	Increased	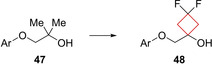	MCHR1 inhibitor^[34]^	Obesity
	Increased, cyclobutyl slows esterase cleavage	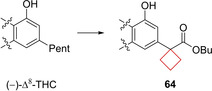	CB1 & CB2 agonist^[36]^	Inflammation
Aryl isostere	Simulates shape and aryl distances well	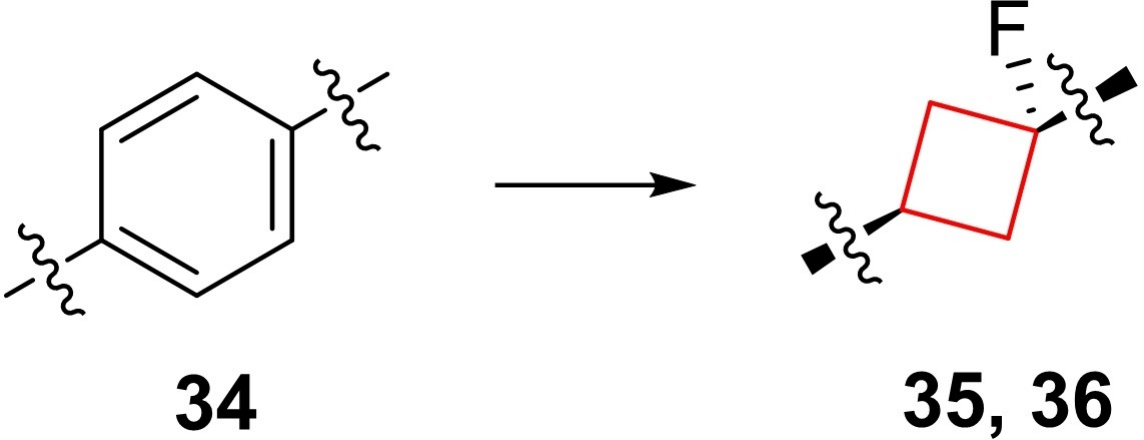	H_3_ receptor antagonist^[37]^	ADHD, AD, narcolepsy
Conformational restriction	Rigid linker increases binding efficiency	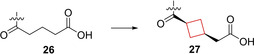	RORγt inverse agonist^[38]^	Rheumatoid arthritis, psoriasis
	Rigid linker increases binding efficiency	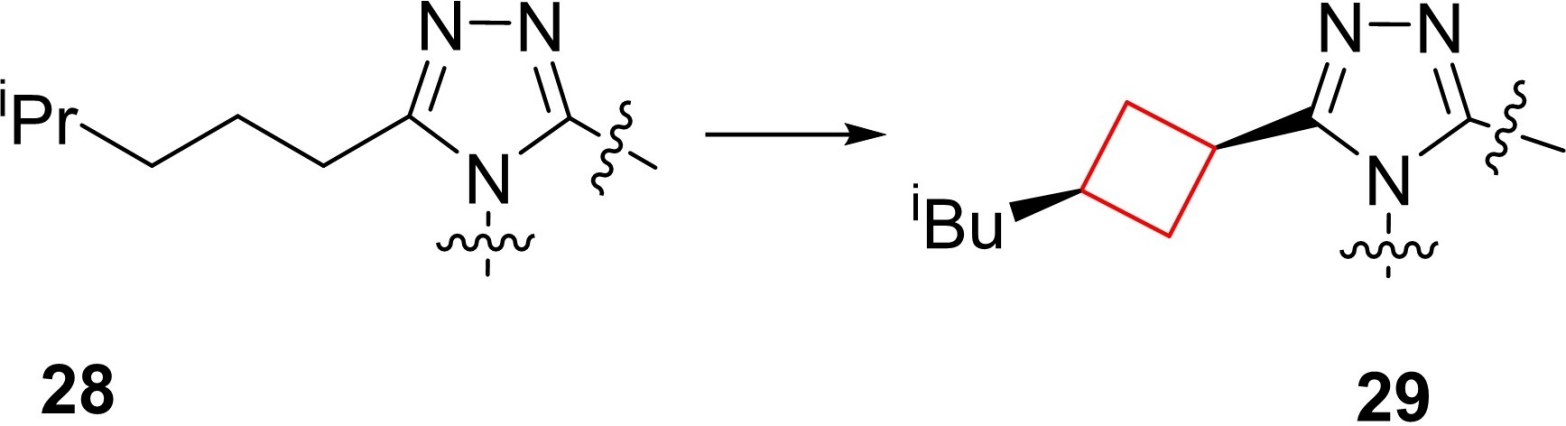	RORγt inhibitor^[39]^	Rheumatoid arthritis, psoriasis
	Cyclization and reduction of degrees of freedom improves PK profile	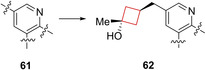	GK activator^[40]^	Type 2 Diabetes
	Increase in rigidity improves TI	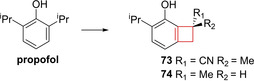	GABA_A_ receptor substrate^[41]^	Anaesthesia
Reduction of planarity	Decreases crystal lattice energy, resulting in faster dissolution	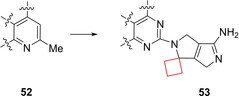	CD38 inhibitor^[42]^	Obesity
Potency alteration	Increased	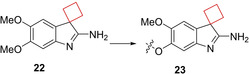	G9a inhibitor^[64]^	Cancer
		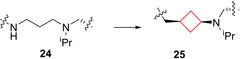	DOT1L inhibitor^[65]^	Cancer
	Increased	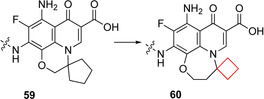	GSK‐3β inhibitor^[108]^	Type 2 Diabetes
Binding affinity alteration	Increased	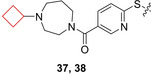	H_3_ receptor antagonist^[81]^	ADHD, AD, narcolepsy
Binding affinity	Increased	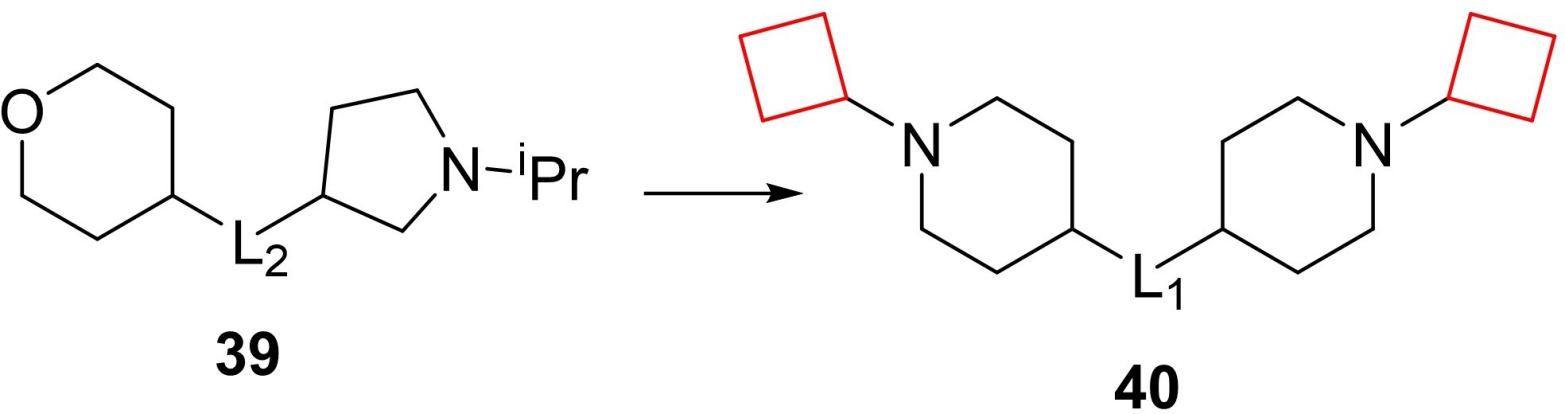	H_3_ receptor antagonist^[82]^	ADHD, AD, narcolepsy
Selectivity	Increased	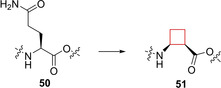	Y_4_R agonist^[99]^	Obesity
Absorption and plasma levels	Increased	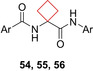	NS5B Thumb pocket 1 inhibitor^[102, 103]^	Hepatitis C
		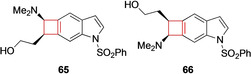	5‐HT6R antagonists^[117]^	Schizophrenia
Cytotoxicity	Lowered	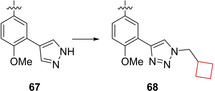	Anti‐leishmanial^[120]^	Visceral Leishmaniasis
Mycobacterium toxicity	Increased	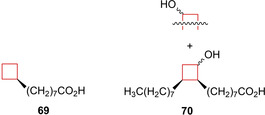	Anti Mtb and Map mycobacterials[^124^, ^125^]	TB

Even though commercial availability and improved synthesis methods have increased the accessibility of cyclobutane rings, it is still not the easiest moiety to include in a small‐molecule drug candidate, especially if the cyclobutyl needs to be incorporated into the core structure of the drug candidate. Therefore, it might not be applied as much compared to other motifs in drug development. However, its unique structure and properties as demonstrated in this review and continuous efforts in synthesis methods might render it a prominent member in the medicinal chemist's toolbox in the not‐too‐distant future.

## List of abbreviations

AD: Alzheimer's disease
ADC: antibody‐drug conjugate
ADHD: attention deficit hyperactivity disorder
ADME: absorption distribution metabolism excretion
ADP: adenosine di‐phosphate
Ala: alanine
apoE: apolipoprotein E4
AR: adrenergic receptor
ARE: antioxidant responsive element
Arg: arginine
Asn: asparagine
Asp: aspartic acid
BACE1: β‐site amyloid precursor protein cleaving enzyme
BBB: blood‐brain barrier
cADPR: cyclic ADP‐ribose
CB1: cannabinoid 1
Cba: cyclobutylanaline
CD38: cluster of differentiation 38
Cit: citrulline
CL: clearance
CNS: central nervous system
CPE: phenotypic cell protection
CYP: cytochrome P450
D3R: dopamine receptor 3
DNA: deoxyribonucleic acid
EHMT2: euchromatic histone methyltransferase 2
EM: electron microscopy
FRET: fluorescence resonance energy transfer
Fsp^3^
: fraction saturated carbons
FTO: fat mass and obesity associated protein
GABA_A_
: gamma aminobutyric acid A
GK: glucokinase
GLN: glutamine
GS: glycogen synthase
GSK‐3β: glycogen synthase kinase 3β
H_3_R: histamine receptor 3
HCV: hepatitis C virus
hERG: human ether‐a‐go‐go‐related gene
HHC: hexahydrocannabinol
HLM: human liver microsomal clearance
HMT: histone methyltransferase
HTS: high throughput screening
HUVEC: human umbilical vein endothelial cells
IVMN: *in vitro* micronucleus
JAK1: janus kinase 1
KEAP: kelch‐like ECH associated protein
LAT: large amino acid transporter
LE: ligand efficiency
Leu: leucine
LOAD: late‐onset Alzheimer's disease
*Map*
: *mycobacterium avium paratuberculosis*
MCH: melanin concentrating hormone
MCHR1: highly efficacious melanin concentrating hormone receptor 1
min: minutes
MLL: mixed lineage leukaemia
*Mtb*
: *mycobacterium tuberculosis*
NAD: nicotinamide adenine dinucleotide
NPY: neuropeptide
NRF2: nuclear factor erythroid‐derived 2
NSB5: nonstructural protein 5B
OAB: overactive bladder
PDE: phosphodiesterase
PET: positron emission tomography
PK: pharmacokinetic
PL: phospholipidosis
PP: pancreatic polypeptide
PROS: PIK3CA‐related overgrowth spectrum
RLM: rat liver microsomal clearance
RNA: ribonucleic acid
RORγt: nuclear receptor retinoic acid receptor‐related orphan receptor gamma
SAR: structure activity relationship
Ser: serine
TB: tuberculosis
THC: tetrahydrocannabinol
TI: therapeutic index
Trp: tryptophan
TTK: threonine tyrosine kinase
Tyr: tyrosine
UV: ultraviolet
Val: valine

## Conflict of interest

The authors declare no conflict of interest.

## Biographical Information

*Marnix R. van der Kolk obtained his MSc in Chemistry cum laude from Radboud University in 2021. During his master's work he performed research on synthetic organic pigments at Radboud University, and was also involved in the development of base metal catalyzed reactions in the Cardenas research group at the Universidad Autónoma de Madrid. He is currently working on a medicinal chemistry project at Radboud University*.

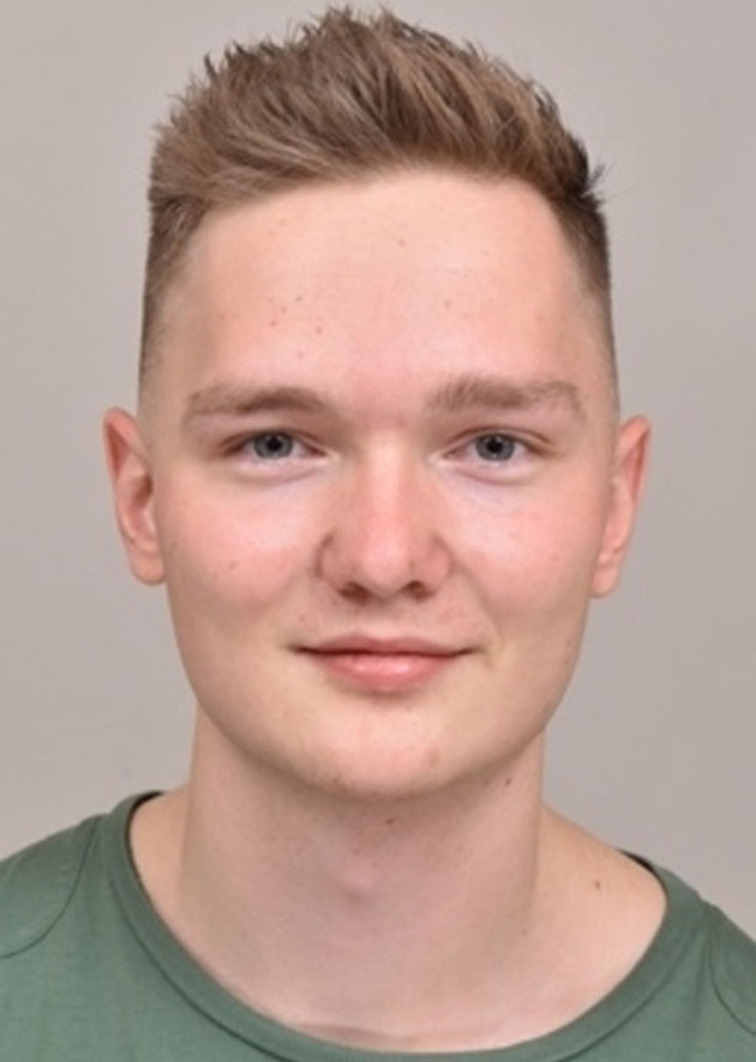

## Biographical Information

*Mathilde A. C. H. Janssen received her MSc in Chemistry from Radboud University in 2020. During her master's work she focused on carbohydrate inhibitors at Radboud University as well as covalent inhibitors at Acerta Pharma B.V. (member of the AstraZeneca group). Currently, she is a PhD candidate working on an NWO TTW project; her goal is to generate novel 3D fragments by using unique synthetic chemistry methodologies such as high‐pressure chemistry and flow chemistry*.

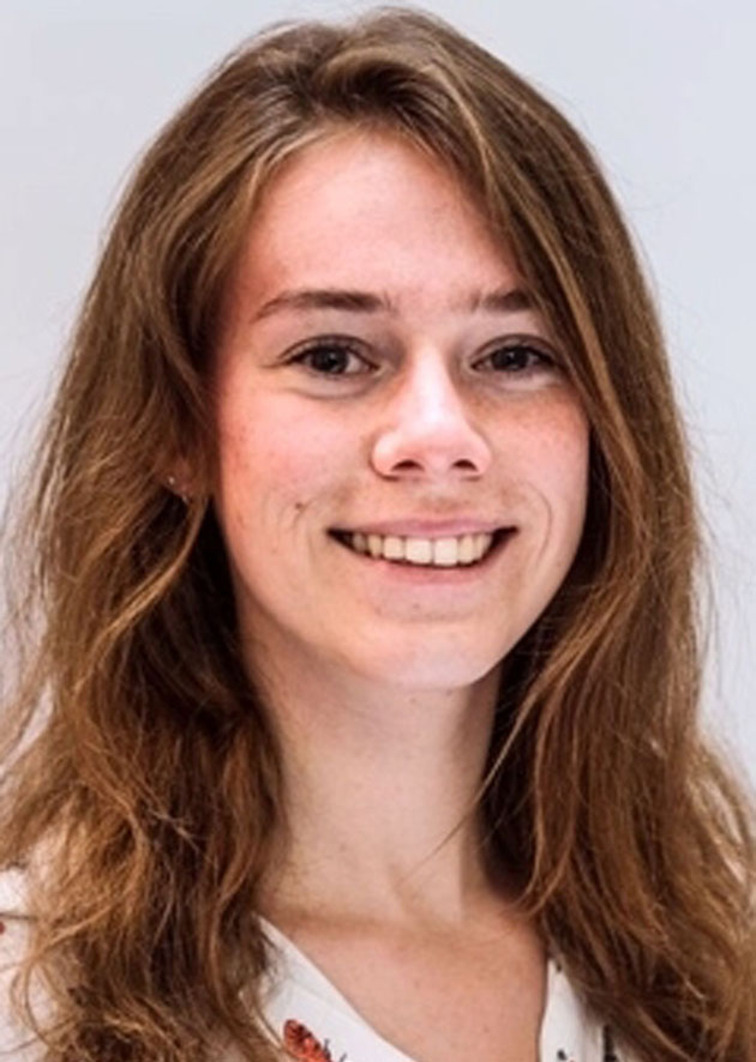

## Biographical Information

*Floris P. J. T. Rutjes received his PhD from the University of Amsterdam in 1993 with Profs. W. N. Speckamp and H. Hiemstra and conducted postdoctoral research in the group of Prof. K. C. Nicolaou at The Scripps Research Institute, La Jolla (USA). In 1995 he was appointed assistant professor in Amsterdam, and in 1999 he became full professor in organic synthesis at Radboud University. Awards include the Gold Medal of the Royal Netherlands Chemical Society (KNCV, 2002), the AstraZeneca award for research in organic chemistry (2003), and Most Entrepreneurial Scientist of the Netherlands (2008). He is currently director of the Institute for Molecules and Materials at Radboud University and President of the European Chemical Society (EuChemS)*.

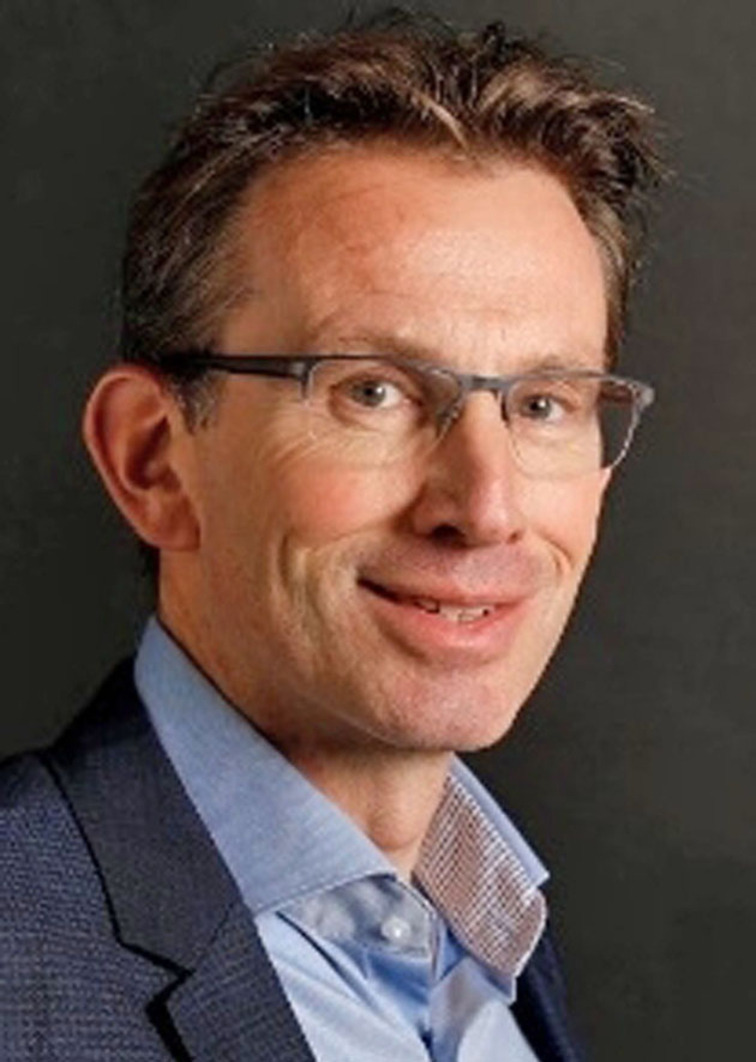

## Biographical Information

*Daniel Blanco Ania began his independent career as a teacher at Academia Blanco (his own private school of Organic Chemistry) in 1994. He then received his PhD from Radboud University (Nijmegen) in 2009 with Prof. F. P. J. T. Rutjes and Dr. Hans W. Scheeren. After his PhD, he conducted postdoctoral research at Radboud University and at the Spanish National Research Council. In 2016, he became staff scientist at Radboud University. In 2019, he was awarded the “Golden Teacher of the Year” award of the Royal Netherlands Chemical Society (KNCV). His research interests include the use of metal‐ and organocatalysis in organic synthesis, photochemistry, flow chemistry, and crop protection*.